# Maladaptive Peripheral Ketogenesis in Schwann Cells Mediated by CB_1_R Contributes to Diabetic Neuropathy

**DOI:** 10.1002/advs.202414547

**Published:** 2025-01-30

**Authors:** Weizhen Li, Tuo Yang, Ningning Wang, Baolong Li, Chuikai Meng, Kaiming Yu, Xiongyao Zhou, Rangjuan Cao, Shusen Cui

**Affiliations:** ^1^ Department of Hand and Foot Surgery China‐Japan Union Hospital of Jilin University Changchun 130033 China; ^2^ Key Laboratory of Peripheral Nerve Injury and Regeneration of Jilin Province Changchun 130033 China

**Keywords:** cannabinoid type 1 receptor, diabetic peripheral neuropathy, ketogenesis, metabolic reprogramming, schwann cell

## Abstract

Diabetic peripheral neuropathy (DPN) is the most common complication of diabetes. Although studies have previously investigated metabolic disruptions in the peripheral nervous system (PNS), the exact metabolic mechanisms underlying DPN remain largely unknown. Herein, a specific form of metabolic remodeling involving aberrant ketogenesis within Schwann cells (SCs) in streptozotocin (STZ)‐induced type I diabetes mellitus is identified. The PNS adapts poorly to such aberrant ketogenesis, resulting in disrupted energy metabolism, mitochondrial damage, and homeostatic decompensation, ultimately contributing to DPN. Additionally, the maladaptive peripheral ketogenesis is highly dependent on the cannabinoid type‐1 receptor (CB_1_R)‐Hmgcs2 axis. Silencing CB_1_R reprogrammed the metabolism of SCs by blocking maladaptive ketogenesis, resulting in rebalanced energy metabolism, reduced histopathological changes, and improved neuropathic symptoms. Moreover, this metabolic reprogramming can be induced pharmacologically using JD5037, a peripheral CB_1_R blocker. These findings revealed a new metabolic mechanism underlying DPN, and promoted CB_1_R as a promising therapeutic target for DPN.

## Graphical abstract

This study demonstrates the maladaptive peripheral ketogenesis in Schwann cells following diabetes mellitus, which serves as a contributor to diabetic peripheral neuropathy. The authors further propose cannabinoid type‐1 receptor in Schwann cells as a promising therapeutic target and JD5037 as a pharmacological intervention to reprogram Schwann cell metabolism.

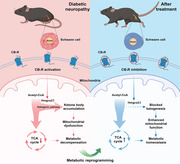

## 1 Introduction

The global prevalence of diabetes mellitus (DM) is estimated to be 540 million, as reported by the International Diabetes Federation in October 2024. Over 50% of this population experiences diabetic neuropathy, which has been recognized as the most prevalent complication of DM.^[^ ^1^ ^]^ As the primary type of diabetic neuropathy and the leading cause of DM‐induced disabling neuropathic pain and lower extremity amputation, DPN affects components throughout the PNS, including the entirety of neurons (from the cell bodies to the terminals) as well as SCs, a type of peripheral glial cell that makes up 80% of the cellular components within the PNS.^[^ ^2^ ^]^ SCs play protective and energy‐supporting roles, thereby endowing the PNS with a short‐term compensatory ability to withstand adverse external conditions.^[^ ^3^ ^]^ However, prolonged exposure to DM results in the metabolic decompensation of SCs, characterized by elevated reactive oxygen species (ROS) production, disrupted mitochondrial function, and inadequate adenosine triphosphate (ATP) generation, ultimately contributing to DPN development.^[^ ^1^, ^2^ ^]^ The specific mechanisms underlying these metabolic alterations in SCs remain unclear, making it difficult to manipulate SC metabolism for therapeutic purposes.

The CB_1_R is a G protein‐coupled receptor highly expressed in the nervous system, particularly the brain.^[^ ^4^ ^]^ This receptor is widely involved in regulating energy homeostasis under various physiological or pathological conditions.^[^ ^5^ ^]^ Although it has been reported that CB_1_R is also expressed in the PNS, its exact metabolic role in the PNS, particularly in the SCs, has not yet been defined.^[^ ^6^ ^]^ In the present study, we identified the specific metabolic remodeling of SCs following STZ‐induced type I diabetes mellitus (T1DM), which is mediated by SC‐CB_1_R and its downstream target Hmgcs2, a key enzyme involved in ketogenesis.^[^ ^7^ ^]^ Ketone bodies, traditionally recognized as alternative energy sources primarily produced by the liver, were found to have neuroprotective effects, thereby contributing to the maintenance of nervous system homeostasis.^[^ ^7^, ^8^ ^]^ However, the involvement of ketone body metabolism in the pathological processes of the nervous system, particularly the PNS, has rarely been reported. Notably, the majority of existing studies on ketone body metabolism, including those related to the nervous system, were conducted within the context of hepatic ketogenesis.^[^ ^9^ ^]^ While a limited number of studies have indicated that vital organs with high metabolic demands, such as the heart and kidneys, possess the potential for extrahepatic ketogenesis under some certain pathological conditions,^[^ ^10^ ^]^ the ketogenic capacity of peripheral tissues, particularly peripheral nerves, and its roles remain poorly characterized in both physiological and pathological conditions. In this study, multiple lines of evidence provided by loss‐of‐function investigations, proteomic analyses, and in vitro studies indicated that the metabolic remodeling of SCs following DM involves aberrant peripheral ketogenesis. We further demonstrated that the PNS could not adapt to this aberrant ketogenesis, resulting in a cascade of maladaptive metabolic disorders ultimately culminating in DPN. Silencing SC‐CB_1_R reversed the metabolic remodeling of SCs by blocking aberrant ketogenesis, resulting in rebalanced metabolic homeostasis of SCs, reduced histopathological changes of the PNS, and improved DPN symptoms. Finally, we showed that the intragastric administration of JD5037, a peripherally restricted (non‐brain‐penetrant) CB_1_R inverse agonist that avoids central side effects,^[^ ^11^ ^]^ pharmacologically induced metabolic reprogramming of SCs, resulting in a significant deceleration of DPN progression. These findings reveal maladaptive peripheral ketogenesis as a previously unrecognized key mechanism underlying DPN, and provide a promising therapeutic target for the prevention and management of DPN.

## 2 Results

### 2.1 DM Triggers Metabolic Remodeling of SCs

The prevalence of DPN among patients with diabetes exceeds 50%, and glycemic control alone is insufficient to completely prevent DPN development.^[^ ^1^, ^12^ ^]^ However, the high incidence and ineffectiveness of glucose control in preventing DPN indicates that this condition cannot be solely attributed to the PNS damage caused by hyperglycemia and hyperosmolarity. Therefore, we hypothesized that there may be previously unidentified specific metabolic mechanisms in the PNS underlying DPN.

**Figure 1:**
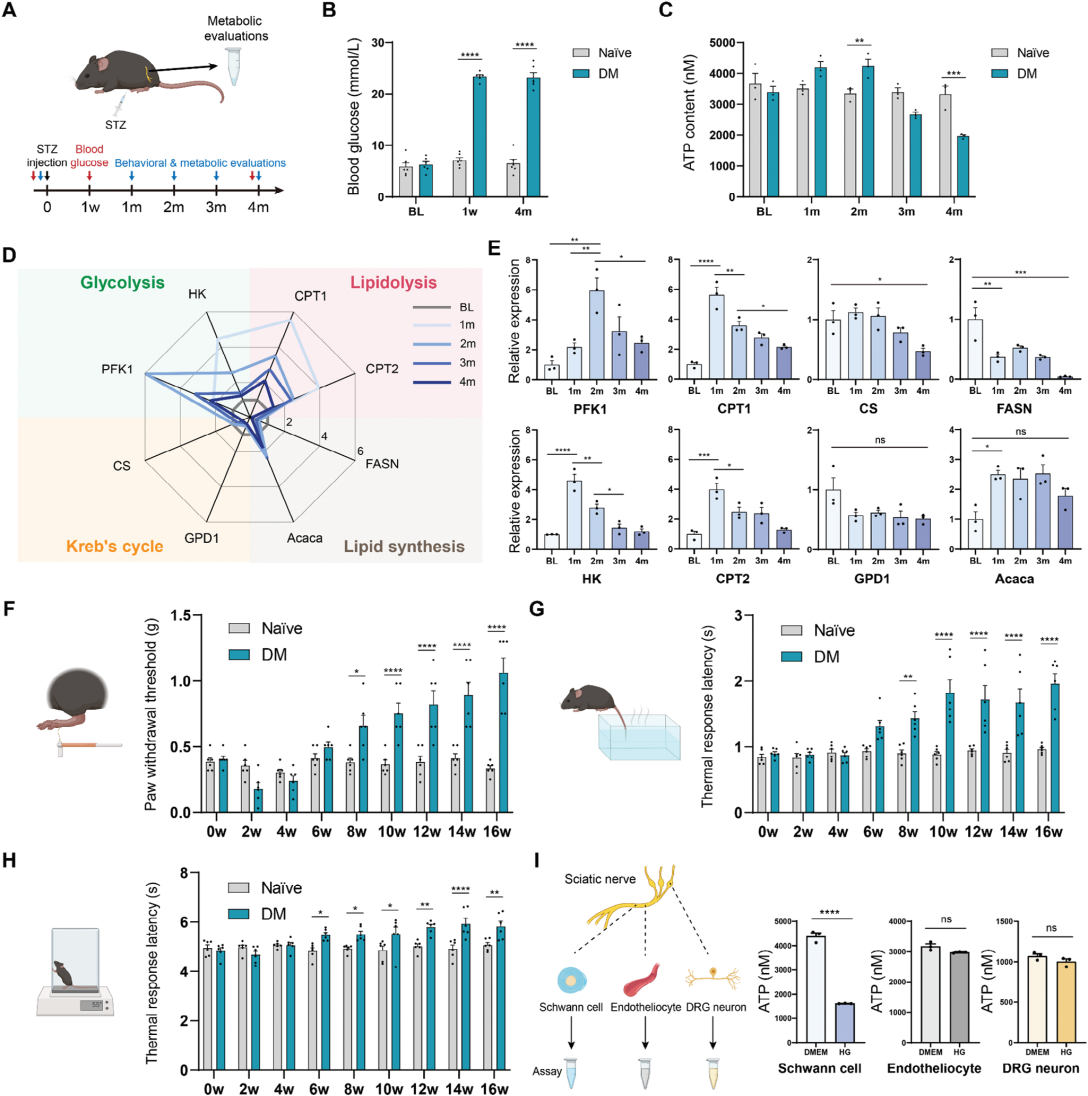
DM‐induced dramatic metabolic remodeling in SCs. A) Schematic presentation of the timeline of STZ injection, behavioral tests, and metabolic evaluations. B) Blood glucose levels were measured one week after STZ injection and before the final sacrifice. n = 6 per group. C) ATP content of the sciatic nerve and its terminals. n = 3 per group. D,E) Summary D) and detailed diagram E) illustrating multiple crucial genes involved in glycolysis, lipolysis, fatty acid synthesis, and the TCA cycle, as evaluated by real‐time PCR. β‐actin was used as a loading control. The BL expression level was standardized to 1 (D). n = 3 per group. F–H) Behavioral tests of DM mice, including the von Frey F), tail‐flick G), and hot plate H) tests. n = 6 per group. I) Intracellular ATP evaluation of three major cellular components of the PNS, DRG neurons, endotheliocytes, and Schwann cells, cultured in standard DMEM (control) or HG (75 mM) for 24h. n = 3 per group. All data are expressed as mean ± SEM. Each data point represents an individual mouse. Statistical comparisons were conducted with two‐way ANOVA followed by Bonferroni's post hoc test (B, C, F‐H); one‐way ANOVA followed by Tukey's post hoc test (E); and two‐tailed t‐test (I). *P < 0.05, **P < 0.01, ***P < 0.001 and ****P < 0.0001.

To explore these potential metabolic mechanisms, we initially established an STZ‐induced T1DM mouse model (**Figure** **1**A,B), in which we characterized ATP production within the sciatic nerve and its terminals, based on the knowledge that failure in ATP production is traditionally recognized to be responsible for DPN.^[^ ^13^ ^]^ Interestingly, we observed that ATP production in the sciatic nerve did not exhibit any immediate decrease following DM, but was maintained for over 2 months, eventually showing a significant decline by 4 months (Figure 1C), which might indicate the existence of compensatory ATP production. To identify the metabolic mechanisms for this compensatory ATP production, we systematically evaluated the expression of a series of key enzymes related to energy metabolism in the sciatic nerve of diabetic mice. Dramatic metabolic remodeling involving robust catabolic activity was observed, characterized by significantly upregulated expression of glycolytic enzymes (HK and PFK1) and lipolytic enzymes (CPT1 and CPT2), particularly within the first 2 months after DM (Figure 1D,E), which coincided with the compensatory ATP production. These findings indicate that the PNS undergoes metabolic remodeling following DM, involving a transition from compensatory ATP production to metabolic decompensation. We subsequently profiled the progression of neuropathic symptoms in diabetic mice, and found that the onset of neuropathic symptoms significantly preceded the decrease in ATP production (Figure 1F–H). This finding strongly indicates that, in addition to ATP deficiency, there exist other key metabolic mechanisms that underlie these neuropathic symptoms during the metabolic remodeling. To determine the exact cellular components of the sciatic nerve undergoing this metabolic remodeling, three key cell types (neurons, SCs, and endothelial cells) were simultaneously cultured and examined for ATP production under HG conditions. SCs were the first to exhibit energy metabolism decompensation as evidenced by a significant reduction in ATP production upon HG stimulation for 24 h, whereas the other two cell types remained unaffected (Figure 1I). We further profiled the ATP alterations in primary SCs under HG conditions. Consistent with the in vivo data based on sciatic nerves, we observed that SCs exhibited an increase in ATP production within 6 h of HG treatment, followed by a decrease at 12 h, and a pronounced energy deficit at 24 h (Figure S1A, Supporting Information). Given that SCs constitute 80% of the cellular components in the PNS, and play a crucial role in energy support,^[^ ^2^, ^14^ ^]^ these data suggest that SCs undergo drastic metabolic remodeling following DM.

### 2.2 SC‐CB_1_R Knockout Attenuates DPN

Given that SCs metabolically support axonal function and that the metabolic dysfunction of SCs greatly contributes to DPN by reducing fuel supply, transferring neurotoxic substrates, and causing myelin degeneration, and considering that SCs exhibit a markedly higher sensitivity to elevated glucose levels compared to other cellular components of peripheral nerves, we hypothesized that the metabolic remodeling of SCs may contribute to the development of DPN and were interested in manipulating SC metabolism for therapeutic purposes.^[^ ^1^, ^14^ ^]^ Since CB_1_R has been found to be extensively involved in CNS metabolism, we proposed CB_1_R as a potential regulator of SC metabolism.^[^ ^4^, ^5^ ^]^ Immunostaining for CB_1_R in the sciatic nerve revealed that this receptor was primarily expressed in the SCs rather than in the axons (Figure S1B, Supporting Information). We also found significantly upregulated expression of CB_1_R in the sciatic nerve of diabetic mice compared to naïve mice (Figure S1C, Supporting Information), indicating a potential role of SC‐CB_1_R in DPN development.

**Figure 2:**
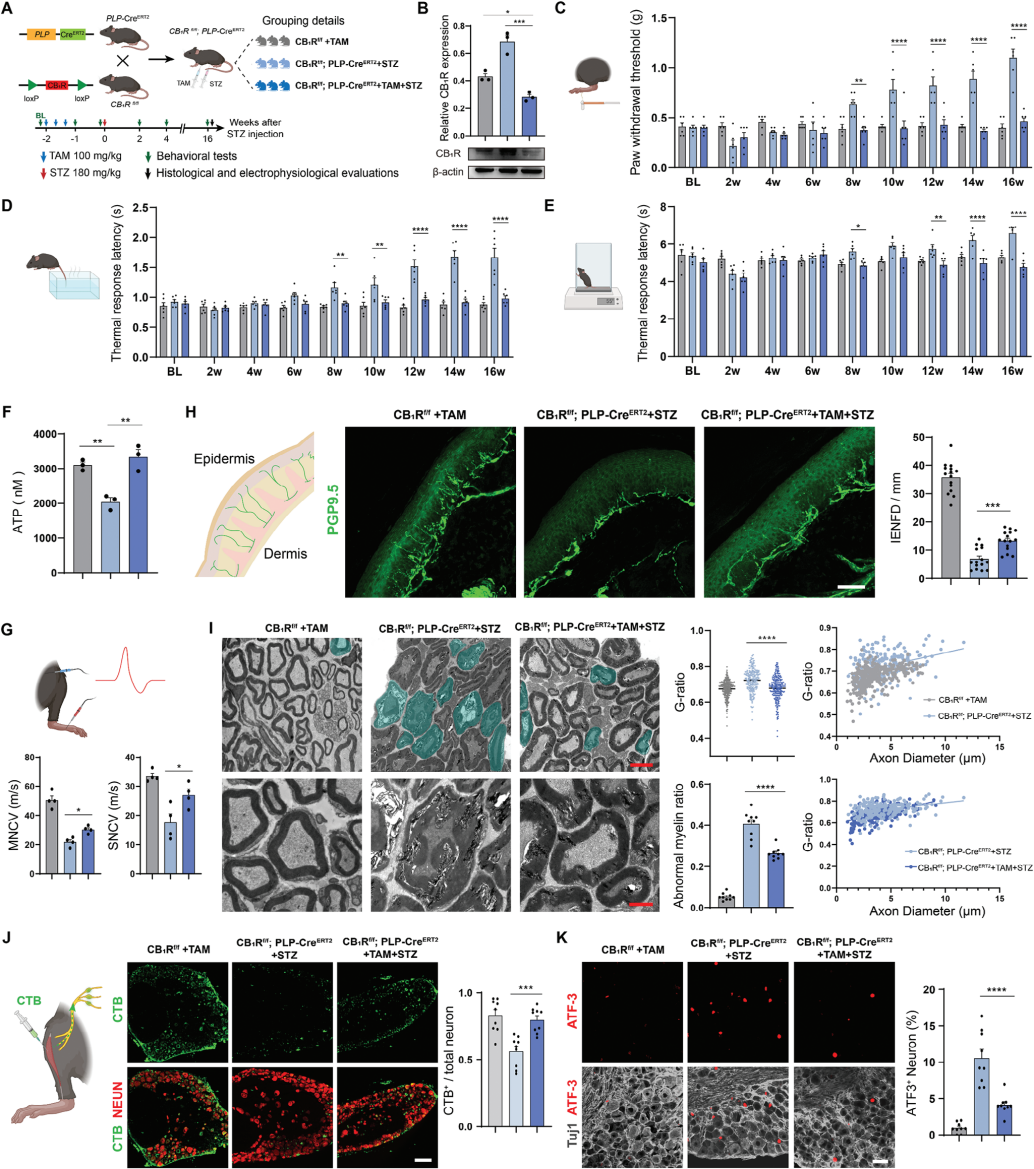
SC‐CB_1_R knockout attenuates DPN. A) Schematic representation of the preparation of transgenic mice, experimental timeline, and grouping information. B) Western blot and quantification of CB_1_R in the sciatic nerves with β‐actin used as a loading control. n = 3 per group. C–E) Behavioral tests, including the von Frey C), tail‐flick D), and hot plate E) tests. n = 6 per group. F) ATP content of the sciatic nerve at 4 months post‐STZ injection. n = 3 per group. G) Quantification of motor and sensory nerve conduction velocities at 4 months post‐STZ injection. n = 4 per group. H) Representative images and quantification of PGP 9.5‐labeled intraepidermal nerve fibers in the hind paws at 4 months post‐STZ injection. Scale bar, 50 µm. n = 15 slices from 5 mice. I) Representative electron micrographs and quantification of the myelin sheaths at 4 months post‐STZ injection. Abnormal myelin sheaths are indicated in viridis. Scale bar, 5 µm (top), 2 µm (bottom). For the g‐ratio, each data point indicates an individual myelin sheath. n = 9 slices from 3 mice. J) Representative images and quantification of the CTB traced DRG neurons at 4 months post‐STZ injection. n = 9 slices from 3 mice. Scale bar,100 µm. K) Representative images and quantification of the ATF3 staining in the affected DRGs at 4 months post‐STZ injection. n = 9 slices from 3 mice. Scale bar, 50 µm. All data are expressed as mean ± SEM. Each data point represents an individual mouse B–G). Statistical comparisons were conducted with one‐way ANOVA followed by Tukey's post hoc test B,F–K); and two‐way ANOVA followed by Bonferroni's post hoc test (C‐E). *P < 0.05, **P < 0.01, ***P < 0.001 and ****P < 0.0001.

To further investigate the role of SC‐CB_1_R in DPN, we crossed CB_1_R*^fl/fl^* with PLP‐Cre^ERT‐2^ mice to create CB_1_R*^fl/fl^*; PLP‐Cre^ERT‐2^ mice, and then conditionally knocked out SC‐CB_1_R through tamoxifen (TAM) injection (**Figure** **2**A,B; Figure S1D,E, Supporting Information). First, to exclude the effect of TAM injection and SC‐CB_1_R knockout on the behavioral baseline of experimental mice, several behavioral tests were performed at 1 and 2 weeks after TAM injection. Overall, we found no significant differences in mechanical or thermal nociception among the three groups (Figure S1F, Supporting Information), indicating that TAM injection and SC‐CB_1_R knockout did not significantly affect the sensory baseline. Additionally, blood glucose levels were assessed prior to STZ injection, 1 week after STZ injection, and before sacrifice to exclude outliers, thereby ensuring the reliability and homogeneity of STZ‐induced T1DM (Figure S2A, Supporting Information). Neuropathic symptoms were evaluated following STZ injection. The data for both mechanical and thermal thresholds indicated that SC‐CB_1_R knockout significantly alleviated the neuropathic symptoms of DPN (Figure 2C–E). For motor function, we assessed the muscle strength of the hind limbs and found that DM significantly impaired muscle contraction intensity in response to 50–125 Hz stimulations,^[^ ^15^ ^]^ which could be rescued by SC‐CB_1_R knockout (Figure S2B, Supporting Information). Next, the ATP content of the sciatic nerve was assessed 4 months following STZ injection, which showed that CB_1_R knockout prevented DM‐induced ATP deficiency (Figure 2F). Motor nerve conduction velocity (MNCV) and sensory nerve conduction velocity (SNCV), both of which are indicators of myelin damage, were subsequently examined through electrophysiological studies, revealing significant improvements in cKO mice (Figure 2G). As decreased intraepidermal nerve fiber density (IENFD) is considered the gold standard for DPN diagnosis,^[^ ^16^ ^]^ IENFD was evaluated by immunostaining for PGP 9.5 in the hind paws. This investigation showed that SC‐CB_1_R knockout attenuated epidermal denervation of the hind paws (Figure 2H). We further assessed the myelin sheath of cKO mice using electron microscopy, and observed that SC‐CB_1_R knockout ameliorated myelin degeneration and reduced the proportion of abnormal myelin sheaths (Figure 2I; Figure S2C, Supporting Information). A G‐ratio analysis based on the axon diameter further indicated that this alleviation covered axons across all diameters (Figure 2I). Additionally, sensory innervation to the muscle was examined by injecting cholera toxin subunit B (CTB) into the tibialis anterior, allowing this toxin to be taken up by nerve terminals and transported to cell bodies.^[^ ^17^ ^]^ Immunostaining of the affected dorsal root ganglia (DRG, L3‐5) revealed a substantial denervation caused by DM, as evidenced by the reduced number of CTB^+^ neurons in the affected DRGs. In cKO mice, denervation was significantly ameliorated following CB_1_R knockout (Figure 2J). Given that DM also severely affects the innervation of motor nerve terminals to the muscles,^[^ ^18^ ^]^ we investigated neuromuscular junction (NMJ) degeneration via NMJ immunostaining and found that the NMJs were significantly disrupted by DM, but rescued by CB_1_R knockout (Figure S2D, Supporting Information).Axonal damage was further evaluated by staining for ATF3, a marker of injured and axotomized neurons, in the affected DRGs. We further found a significant reduction in ATF3^+^ neurons in the L3‐5 DRGs, indicating that CB_1_R knockout alleviates DM‐induced axonal damage (Figure 2K).

Taken together, these findings demonstrate that the inhibition of SC‐CB_1_R reversed the DM‐induced energy metabolic imbalance of SCs, alleviated the histopathological changes of DPN, and ameliorated neuropathic symptoms, indicating that SC‐CB_1_R may play a key role in DM‐induced metabolic remodeling of SCs, thus indicating it as a promising therapeutic target for DPN.

### 2.3 Hmgcs2 is a Key Downstream Target of SC‐CB_1_R

**Figure 3:**
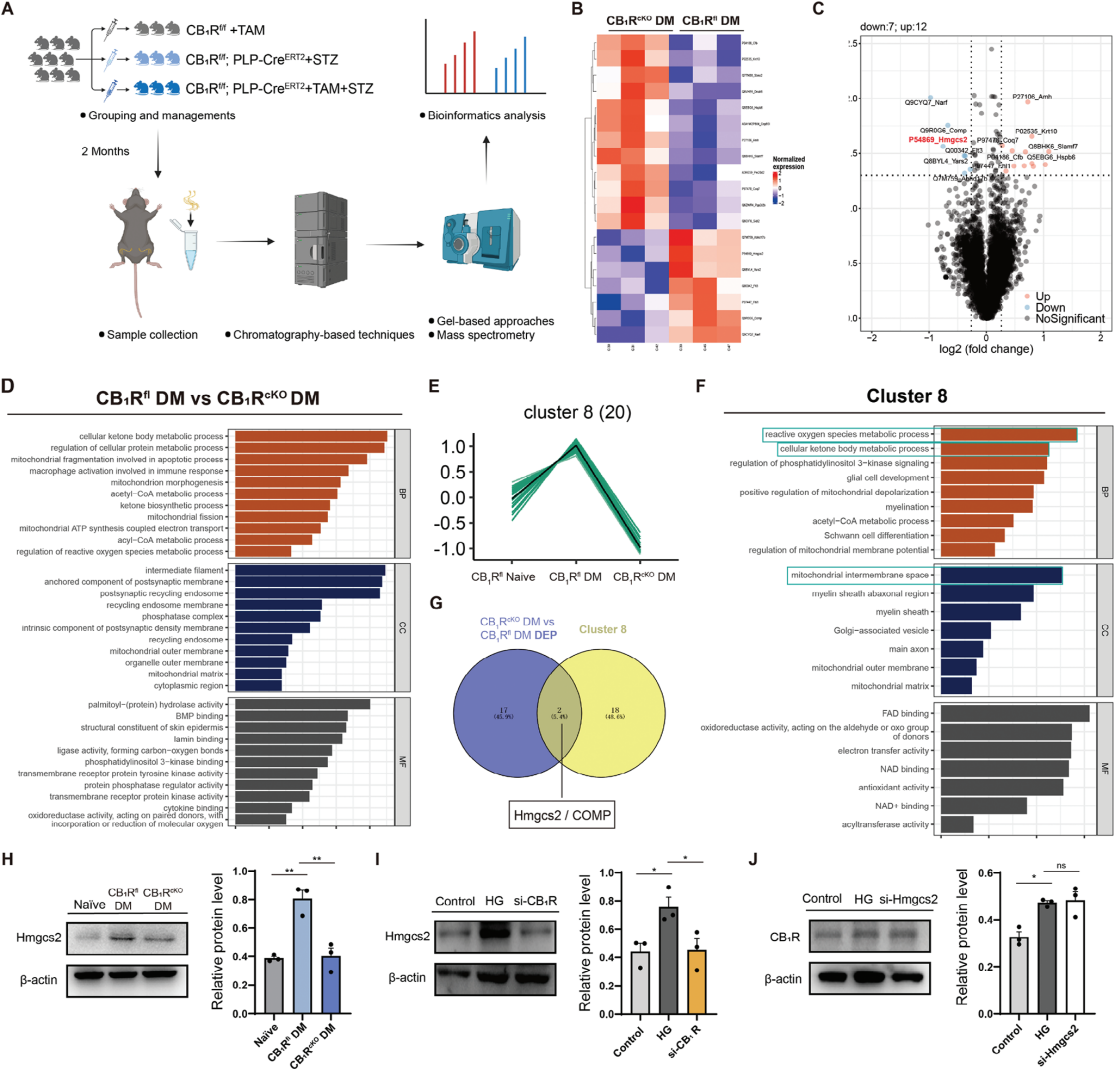
Hmgcs2 is a key downstream target of SC‐CB_1_R. A) Schematic workflow and grouping details for the quantitative proteomic analysis of the sciatic nerve at 2 months post‐STZ injection. Grouping details: CB_1_R^fl^ Naïve (CB_1_R^fl/fl^ + TAM); CB_1_R^fl^ DM (CB_1_R^fl/fl^; PLP‐Cre^ERT2^ + STZ); and CB_1_R^cKO^ DM (CB_1_R^fl/fl^; PLP‐Cre^ERT2^ + TAM + STZ). n = 3 per group. B,C) Heatmap B) and volcano map C) of differential proteins between CB_1_R^cKO^ DM mice and CB_1_R^fl^ DM mice. D) GO analysis of the differential proteins between the CB_1_R^cKO^ DM and CB_1_R^fl^ DM groups. E) Expression pattern of 20 proteins in Cluster 8 identified through Mfuzz analysis. F) GO analysis of proteins in Cluster 8. G) Intersection of differential proteins between the CB_1_R^cKO^ DM and CB_1_R^fl^ DM groups within Cluster 8. H) Western blot analyses and quantifications of Hmgcs2 protein abundance in the sciatic nerves of the 3 groups. n = 3 per group. I) Western blot analyses and quantifications of Hmgcs2 protein abundance in the primary SCs transfected with siRNAs for 72 h, and treated with HG for 24h. Grouping details: standard DMEM (Control), negative siRNA + HG (HG), and CB_1_R siRNA + HG (si‐CB_1_R). n = 3 per group. J) Western blot analyses and quantifications of CB_1_R protein abundance in the primary SCs transfected with siRNAs for 72 h and treated with HG for 24 h. Grouping details: standard DMEM (Control), negative siRNA + HG (HG), and Hmgcs2 siRNA + HG (si‐Hmgcs2). n = 3 per group. All data are expressed as mean ± SEM. Statistical comparisons were conducted with one‐way ANOVA followed by Tukey's post hoc test. *P < 0.05, and **P < 0.01.

To gain mechanistic insight into how SC‐CB_1_R contributes to DM‐induced metabolic remodeling of SCs, we isolated the sciatic nerves and their terminals from SC‐CB_1_R cKO DM mice, littermate DM mice, and littermate naïve mice 2 months following DM for quantitative proteomic analysis to characterize their protein expression during DM‐induced metabolic remodeling (**Figure** **3**A). We further analyzed the differentially expressed proteins between cKO DM mice and littermate DM mice (Figure 3B,C), as well as those between littermate DM mice and littermate naïve mice (Figure S3A,B, Supporting Information). GO analysis of these differentially expressed proteins revealed that SC‐CB_1_R knockout exerted a significant impact on energy metabolism processes of the sciatic nerve in diabetic mice (Figure 3D; Figure S3C, Supporting Information), indicating that CB_1_R may play a role in mediating DM‐induced energy metabolism alterations in SCs. To further elucidate the mechanisms underlying CB_1_R‐mediated alterations in SC energy metabolism, all quantified proteins were subjected to Mfuzz cluster analysis and divided into eight clusters based on their altered expression among the three groups of experimental mice (Figure S3D, Supporting Information). Among these clusters, Cluster 8 was identified as it exhibited coincident changes in expression and showed a high correlation with ROS production and energy metabolism (Figure 3E,F; Figure S3E, Supporting Information). Subsequently, we intersected the differential proteins between cKO DM mice and littermate DM mice within Cluster 8, which showed that Hmgcs2 may function as a key protein involved in CB_1_R‐mediated alterations in SC energy metabolism following DM (Figure 3G). To determine the relationship between Hmgcs2 and CB_1_R, we subsequently examined Hmgcs2 expression in the sciatic nerve, finding that Hmgcs2 was significantly upregulated 2 months after DM but downregulated upon CB_1_R knockout (Figure 3H). In vitro investigation of cultured SCs further revealed increased Hmgcs2 expression in SCs under HG conditions (Figure S3H, Supporting Information), indicating a potential role for Hmgcs2 in DM‐induced SC metabolic alterations. Additionally, the inhibition of CB_1_R by CB_1_R‐siRNA down‐regulated Hmgcs2 expression (Figure 3I), whereas the inhibition of Hmgcs2 exerted no significant impact on CB_1_R expression (Figure 3J). These findings indicate that Hmgcs2 serves as a pivotal downstream target of SC‐CB_1_R and plays a key role in the regulation of SC energy metabolism following DM.

We next investigated how SC‐CB_1_R regulates Hmgcs2 expression. Considering that: 1) KEGG analysis revealed a significant upregulation of genes associated with the PPAR signaling pathway following DM, whereas downregulation was observed following SC‐CB_1_R knockout (Figure S3G, Supporting Information); 2)  Peroxisome proliferator‐activated receptors (PPARs) are a family of ligand‐activated transcription factors, of which PPARα serves as the chief transcription factor, and is extensively involved in ketogenesis;^[^ ^19^ ^]^ notably, the transcription of Hmgcs2 heavily relies on the functioning of PPARα;^[^ ^20^ ^]^ 3) CB_1_R and PPARα are strongly correlated, particularly in the regulation of lipid metabolism and energy balance;^[^ ^21^ ^]^ we hypothesize that PPARα may serve as a key mediator by which SC‐CB_1_R regulates Hmgcs2 expression. To test this hypothesis, we first examined the expression levels of PPARα in the sciatic nerves of the cKO mice. PPARα was found upregulated following DM; however, this upregulation was inhibited by SC‐CB_1_R knockout (Figure S4A, Supporting Information). Silencing CB_1_R by siRNA downregulated the HG‐induced upregulation of PPARα in primary SCs in vitro (Figure S4B, Supporting Information). HG‐cultured SCs treated with GW6471, a PPARα inhibitor, exhibited downregulation of Hmgcs2 (Figure S4C, Supporting Information). Since PPARα activity is regulated by the AMPK‐mTOR pathway, which can be directly activated by CB_1_R,^[^ ^19^, ^22^ ^]^ and our proteomic data showed upregulation of AMPK and mTOR signaling following CB_1_R knockout (Figure S4F,G, Supporting Information), we further examined the activation of this pathway. Data indicated that HG inhibited the activation of AMPK and mTOR, whereas silencing CB_1_R reversed this inhibitory effect (Figure S4D,E, Supporting Information). Together, these findings indicated that SC‐CB_1_R may regulate Hmgcs2 expression through the AMPK‐mTOR‐PPARα pathway.

### 2.4 Maladaptive Ketogenesis in SCs Contributes to DPN

**Figure 4:**
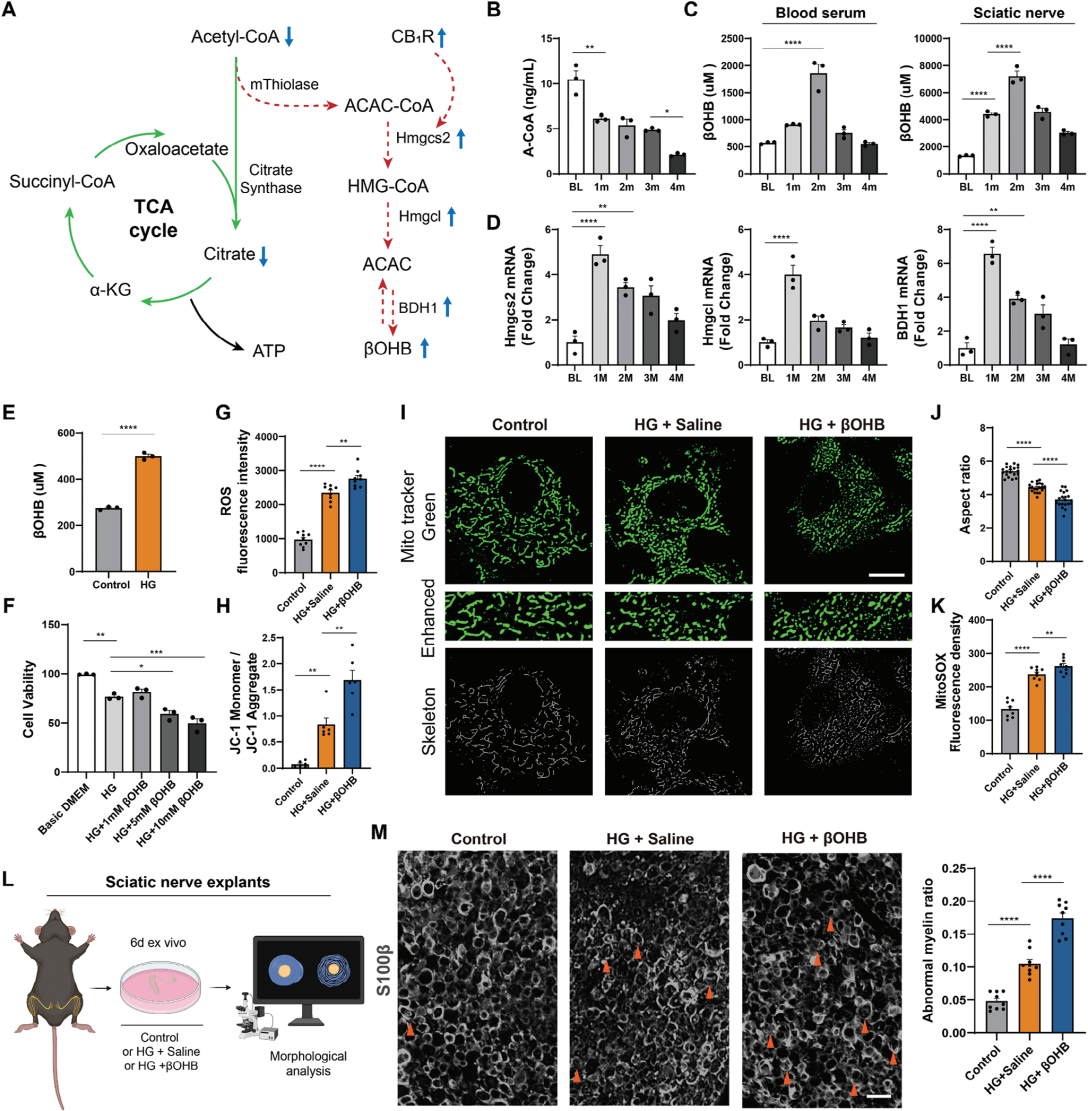
Maladaptive ketogenesis in SCs contributes to DPN. A) Metabolic diagram illustrating the diversion of acetyl‐CoA caused by aberrant ketogenesis during DM‐induced SC metabolic remodeling. B) ELISA for acetyl‐CoA in the sciatic nerve lysates at different time points after DM. n = 3 per group. C) βOHB levels in the sciatic nerve lysates and blood serum. n = 3 per group. D) Real‐time PCR analysis of key genes involved in ketogenesis in the sciatic nerve and its terminals. β‐actin was used as a loading control. n = 3 per group. E) Intracellular βOHB levels in primary SCs treated with or without HG for 24h. n = 3 per group. F) The cell viability of SCs treated with varying concentrations of βOHB for 24 h was assessed by CCK‐8 assay. n = 3 per group. G–K) Intracellular ROS G), mitochondrial membrane potential H), representative images of mitochondrial morphology I), quantification of mitochondrial aspect ratio J), and mitochondrial ROS K) in primary SCs. Scale bar, 10 µm. n = 9 G,K), 6 H), and 3 I,J) per group. Each data point indicates a single mitochondrion (J). L) Schematic of the ex vivo investigation of isolated sciatic nerve explants. M) Representative images of immunostaining for S100β in the sciatic nerve explants, with quantification of the abnormal myelin ratio. Orange arrows indicate abnormal myelin sheaths. Scale bar, 20 µm. n = 9 slices from 3 mice. All data are expressed as mean ± SEM. Statistical comparisons were conducted with one‐way ANOVA followed by Tukey's post hoc test (B‐D, F‐H, J, K, M); and two‐tailed t‐test (E). *P < 0.05, **P < 0.01, ***P < 0.001 and ****P < 0.0001.

After confirming Hmgcs2 as a key downstream target of CB_1_R, we investigated how CB_1_R regulates SC metabolic remodeling via Hmgcs2. Given that 1) Hmgcs2 is a fate‐committing enzyme highly associated with ketogenesis;^[^ ^7^, ^23^ ^]^ 2) our proteomic data revealed an extensive involvement of ketone body metabolism in DM/CB_1_R knockout induced metabolic alterations (Figure 3D,F); and 3) we observed a lack of proportional ATP production in the sciatic nerve within the first two months following DM, despite high levels of catabolic activity (Figure 1C–E), we hypothesized that aberrant ketogenesis occurs in SCs subsequent to DM, resulting in a substantial diversion of acetyl‐CoA, and ultimately disrupting the energy metabolic homeostasis of SCs (**Figure** **4**A).

To address this issue, we initially assessed the levels of acetyl‐CoA, a crucial intermediate in energy metabolism that serves as a shared substrate for both the TCA cycle and ketogenesis. Interestingly, we detected a significant reduction in acetyl‐CoA levels in sciatic nerves following DM (Figure 4B), which contradicted the aforementioned findings of robust catabolic activity (Figure 1G,H), indicating a substantial diversion of acetyl‐CoA into other metabolic pathways. To determine whether the substantial quantity of acetyl‐CoA generated through robust catabolism was diverted toward the ketogenic pathway, we subsequently profiled the alteration of βOHB content in the blood serum and sciatic nerve following DM. Interestingly, we detected significantly upregulated βOHB within the sciatic nerve, reaching a level much higher than that in blood serum (Figure 4C), indicating the possibility that localized ketogenesis was occurring within the nerve. A series of key enzymes involved in the ketogenic pathway, including Hmgcs2, Hmgcl, and BDH1, were further evaluated, revealing marked elevation following DM (Figure 4D). In addition, the peak levels of βOHB and ketogenic enzymes in the sciatic nerve occurred within the first two months following DM, which coincided with the initiation of neuropathological symptoms, as we had observed (Figure 1D–F), indicating that such localized ketogenesis was a potential contributor to DPN development. To specifically assess the aberrant ketogenesis in SCs, we further analyzed SCs cultured in HG, confirming that their βOHB content was much higher than that of control SCs (Figure 4E).

The fact that peripheral nerve tissue does not typically produce ketone bodies raises doubts regarding its capacity to adapt to aberrant ketogenesis, particularly the localized accumulation of ketone bodies. Subsequently, we tested the potential impact of ketone body accumulation on SCs in diabetic conditions by supplementing HG‐cultured SCs with βOHB of three concentrations (1, 5, and 10 mM), according to the previously detected βOHB levels in the sciatic nerve (Figure 4C). The localized accumulation of ketone bodies, including an additional 5 and 10 mM of βOHB, significantly impacted the survival of SCs (Figure 4F). To investigate how ketone body accumulation induces SC damage, we analyzed intracellular ROS levels, detecting an increase in ROS production in SCs following the addition of an extra 10 mM βOHB (Figure 4G). We also found that elevated local concentrations of ketone bodies markedly damaged SC mitochondria, manifesting as abnormal mitochondrial membrane potential, mitochondrial fragmentation, and increased mitochondrial ROS production (Figure 4H–K). Such increased ROS production and mitochondrial damage may significantly disrupt SC homeostasis, leading to myelin destruction. To further confirm the myelin destruction induced by ketone body accumulation, ex vivo investigations were conducted on the isolated sciatic nerve explants, while the morphology of myelin was evaluated (Figure 4L), revealing a marked myelin disruption induced by ketone body accumulation (Figure 4M). Collectively, the above evidence indicates that aberrant ketogenesis occurs in SCs following DM, to which it is difficult for the PNS to adapt. Such maladaptive ketogenesis not only disrupts the energy metabolic homeostasis, but also leads to localized ketone body accumulation, thereby exerting detrimental effects on SCs, and eventually participating in DPN.

### 2.5 Inhibition of SC‐CB_1_R Improves DPN by Blocking Maladaptive Ketogenesis

**Figure 5:**
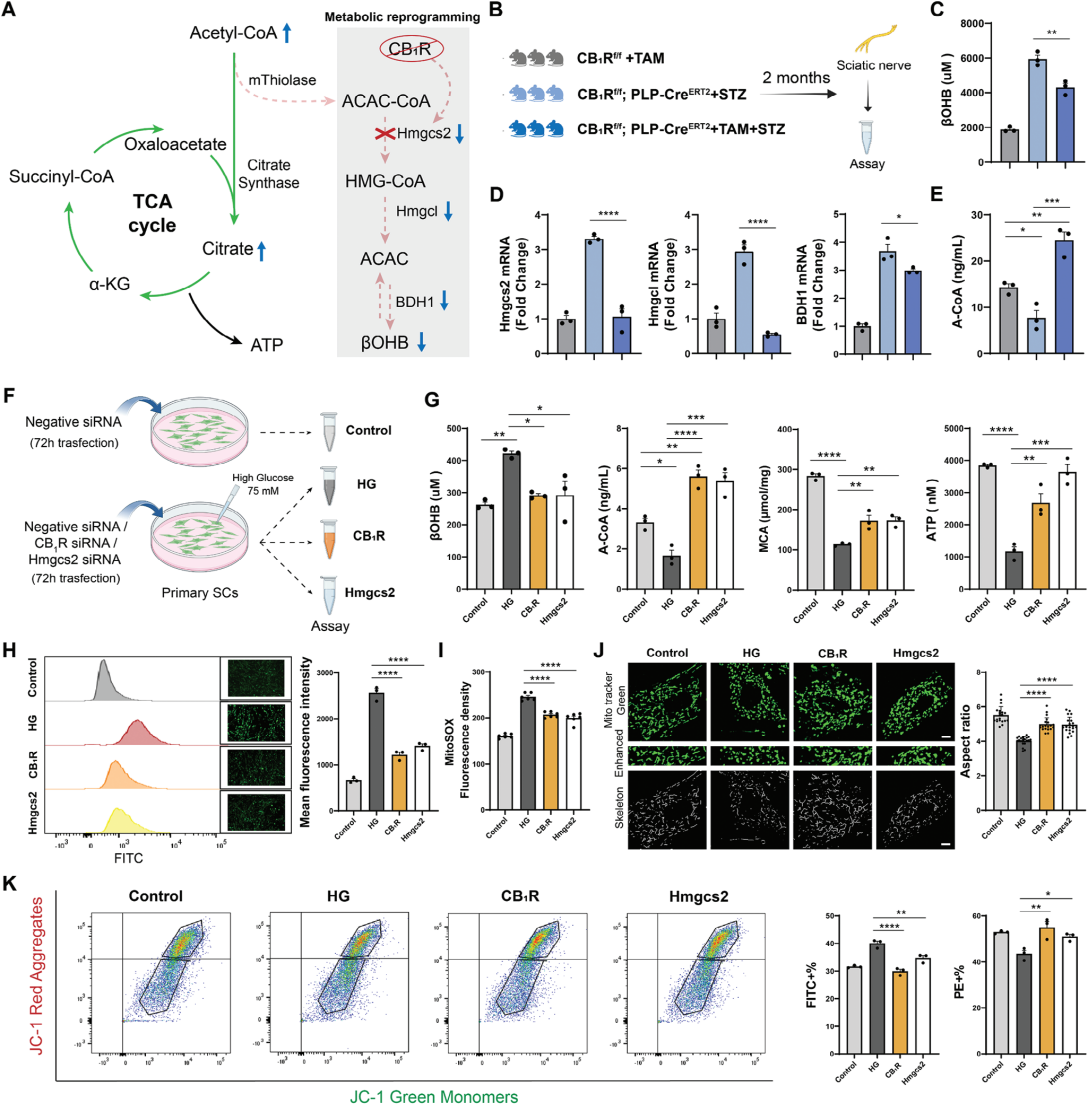
CB_1_R inhibition blocks aberrant ketogenesis, and prevents mitochondrial dysfunction. A) Metabolic diagram illustrating the SC metabolic reprogramming induced by CB_1_R silencing. B) Schematic and grouping details of the evaluations of a series of indicators related to ketogenesis. C) βOHB levels in the sciatic nerves. n = 3 per group. D) Levels of 3 key enzymes involved in ketogenesis in the sciatic nerves. n = 3 per group. E) Levels of acetyl‐CoA in the sciatic nerves. n = 3 per group. F) Schematic and grouping of the in vitro experiment using primary SCs. G) Levels of intracellular metabolites and substrates (intracellular βOHB, acetyl CoA, mitochondrial citric acid, and ATP respectively) of the SCs. n = 3 per group. H) Flow cytometric analysis of intracellular ROS levels, with representative images. n = 3 per group. I) Quantification of MitoSOX evaluated mitochondrial ROS showing levels of mitochondria‐derived ROS in the SCs. n = 6 per group. J) Representative images of Mito tracker Green‐labeled mitochondrial morphology and quantification of the mitochondrial aspect ratio. Scale bar, 5 µm. n = 6, each data point indicates a single mitochondrion. K) Flow cytometric analysis of ΔΨm and quantification of the mitochondria membrane potential. n = 3 per group. All data are expressed as mean ± SEM. Each data point represents an individual mouse. Statistical comparisons were conducted with one‐way ANOVA followed by Tukey's post hoc test. *P < 0.05, **P < 0.01, ***P < 0.001 and ****P < 0.0001.

Next, we investigated the role of CB_1_R in aberrant ketogenesis and analyzed how CB_1_R inhibition improves DPN (**Figure** **5**A). We examined a series of indicators related to ketone body metabolism in the sciatic nerves of SC‐CB_1_R cKO and littermate mice two months post‐DM (Figure 5B). Our results revealed a significant reduction in the levels of βOHB in the sciatic nerve of cKO mice compared with that in the littermate DM mice, indicating a pivotal role of SC‐CB_1_R in DM‐induced SC ketogenesis (Figure 5C). We further evaluated the mRNA levels of three key enzymes involved in ketogenesis and found that they were significantly reduced in the cKO mice (Figure 5D). This finding further supports the notion that SC‐CB_1_R knockout leads to diminished aberrant ketogenesis. As expected, we further detected a substantial rebound in acetyl‐CoA levels in cKO mice (Figure 5E), indicating that aberrant ketogenesis may lead to a significant diversion of acetyl‐CoA, and that blocking this aberrant ketogenesis prevented acetyl‐CoA shunting.

To further investigate the impact of CB_1_R inhibition on SC metabolic remodeling, primary SCs cultured under HG conditions were transfected with negative/CB_1_R/Hmgcs2 siRNA (Figure 5F). Consistent with the above in vivo studies in cKO mice, we detected elevated βOHB, reduced acetyl‐CoA, decreased mitochondrial citrate, and a significant declined ATP production in HG‐cultured SCs compared to control SCs (Figure 5G). In contrast, the inhibition of either CB_1_R or Hmgcs2 effectively suppressed abnormal ketogenesis, thus blocking acetyl‐CoA shunting, increasing mitochondrial citrate levels, and ultimately resulting in a substantial increase in ATP production (Figure 5G). Notably, in both the sciatic nerves of cKO mice and SCs treated with CB_1_R/Hmgcs2 siRNA, blocking aberrant ketogenesis restored acetyl‐CoA to levels higher than those in naïve mice/SCs, which is consistent with the aforementioned observation of robust catabolic activity in the sciatic nerve following DM (Figure 1G,H).

We subsequently investigated whether blocking aberrant ketogenesis could mitigate ROS levels and enhance mitochondrial function by quantifying both intracellular and mitochondrial ROS levels in SCs cultured under HG conditions. These results showed that the inhibition of ketogenesis significantly attenuated the DM‐induced elevation of ROS (Figure 5H,I). Furthermore, the evaluations of mitochondrial health demonstrated that targeting CB_1_R or Hmgcs2 to inhibit abnormal ketogenesis markedly improved mitochondrial function (Figure 5J,K).

Altogether, the above results indicate that the CB_1_R‐Hmgcs2 axis mediates maladaptive ketogenesis in SCs following DM, resulting in a significant diversion of acetyl‐CoA, impairment of the TCA cycle, increased ROS production, mitochondrial dysfunction, and ultimately decompensated homeostasis, resulting in DPN. The inhibition of CB_1_R can reprogram the abnormal metabolic remodeling of SCs, thereby blocking maladaptive ketogenesis and improving DPN (Figure S6, Supporting Information).

### 2.6 Peripheral CB_1_R Antagonist JD5037 Alleviates DPN

Our next objective was to explore the potential therapeutic applications of the pharmacological inhibition of CB_1_R. Considering the difficulty in the clinical promotion of traditional CB_1_R antagonists due to their strong central side effects, our focus was directed toward JD5037 (JD), a peripherally restricted (non‐brain‐penetrant) inverse agonist targeting CB_1_R.^[^ ^24^ ^]^

**Figure 6:**
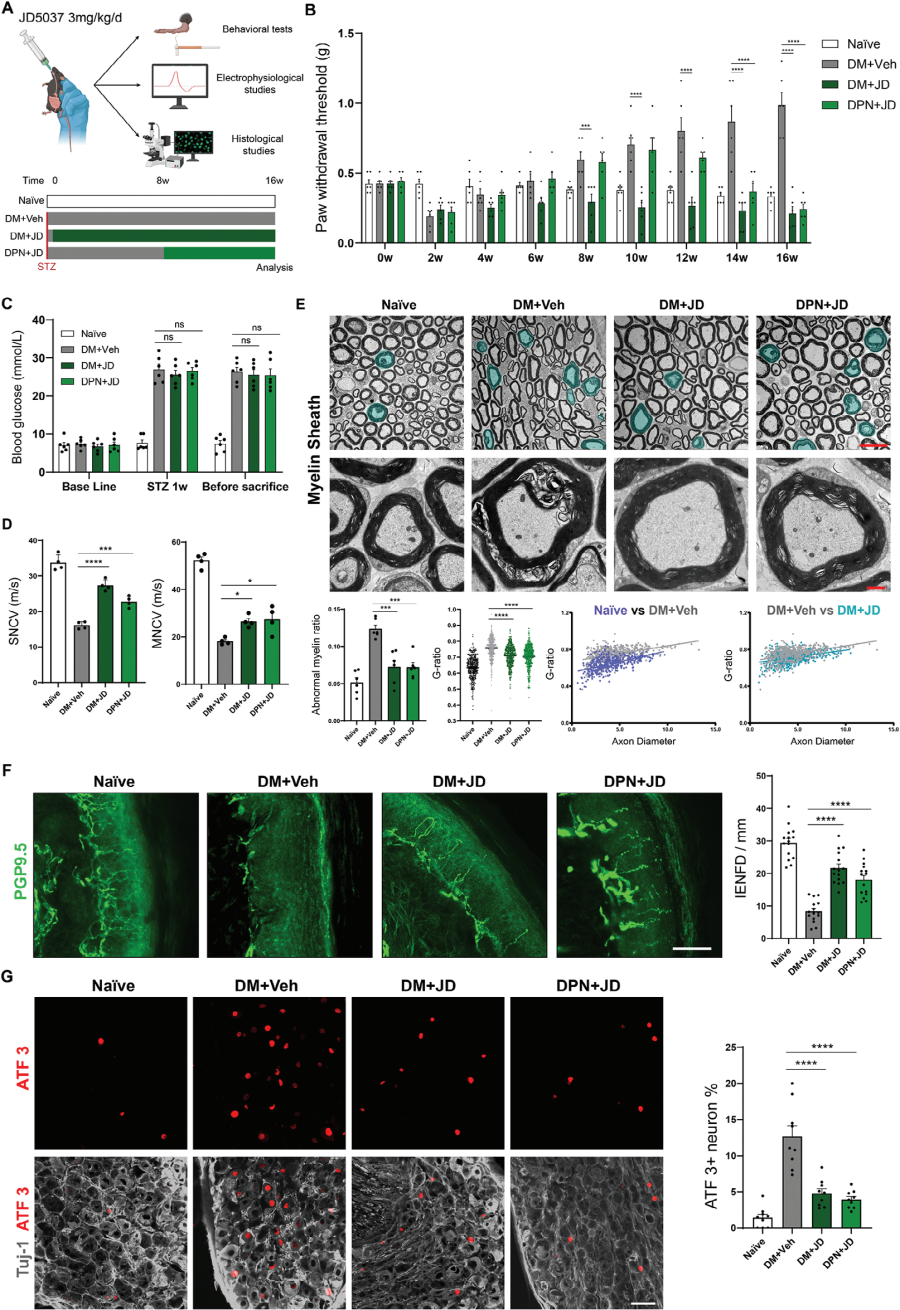
JD5037 attenuates DPN. A) Grouping and timeline for JD administration and the subsequent evaluations. B) Mechanical nociception as evaluated by von Frey test. n = 6 per group. C) Blood glucose assessment. n = 6 per group. D) Electrophysiological evaluations of the nerve conduction velocity at 4 months post‐DM. n = 4 per group. E) Representative electron micrographs of the sciatic nerves and quantifications of the myelin sheath thickness (g‐ratio) and abnormal myelin sheaths. Abnormal myelin sheaths are indicated in viridis. Scale bar, 10 µm (top), 1 µm (bottom). n = 6 per group. F) Representative images and quantification of PGP 9.5‐labeled intraepidermal nerve fibers. Scale bar, 50 µm. n = 15 slices from 3 mice. G) Representative images and quantification of ATF3^+^ neurons in the affected DRGs. Scale bar, 50 µm. n = 9 slices from 3 mice. All data are expressed as mean ± SEM. Statistical comparisons were conducted with two‐way ANOVA followed by Bonferroni's post hoc test B,C) and one‐way ANOVA followed by Tukey's post hoc test (D‐G). *P < 0.05, ***P < 0.001, and ****P < 0.0001.

To investigate the therapeutic effects of JD on DPN, we administrated mice with JD following the onset of DM (DM + JD). Considering that, in clinical scenarios, a significant number of patients do not initiate therapeutic interventions until they exhibit evident neuropathic symptoms, we also established a DPN + JD group in which JD was promptly administrated following the detection of neuropathic symptoms (8 weeks after DM), to determine whether JD could attenuate pre‐existing DPN. A series of behavioral, electrophysiological, and histological studies was subsequently conducted to characterize the therapeutic effects of JD (**Figure** **6**A). Behavioral data revealed that whole‐course administration of JD greatly ameliorated DPN symptoms, while delayed JD treatment also exhibited significant therapeutic effects at 14–16 weeks (Figure 6B; Figure S5A,B, Supporting Information). We further found that JD decreased the levels of βOHB in the sciatic nerves (Figure S5C, Supporting Information). To eliminate the potential impact of the systemic administration of JD on blood glucose, we concurrently assessed the blood glucose levels of JD treated mice, observing no statistically significant differences (Figure 6C), indicating that JD primarily exerted its therapeutic effect by modulating PNS metabolism rather than by controlling blood glucose. Electrophysiological studies further revealed accelerated sensory and motor nerve conduction velocities in JD‐treated mice following both whole‐course and delayed administration (Figure 6D). The myelin sheath was evaluated by electron microscopy (Figure 6E), which revealed that the abnormal myelin and G‐ratios were significantly improved in both the DM + JD and DPN + JD groups. G‐ratio analysis based on axon diameter further revealed that the therapeutic effect of JD covered axons of all tested diameters. In addition, IENFD evaluation showed better peripheral innervation in both of the JD‐treated groups (Figure 6F). Finally, immunofluorescence staining for ATF3 in affected DRGs (Figure 6G), CTB‐traced DRG neurons (Figure S5D, Supporting Information), and NMJs (Figure S5E, Supporting Information) revealed that JD‐treated mice exhibited reduced axonal damage and enhanced innervation. These findings suggest that JD effectively prevents both the occurrence and progression of DNP, highlighting the clinical translational potential of peripheral CB_1_R antagonists as a promising therapeutic approach to prevent and treat DPN.

## 3 Discussion

Traditionally, loss of ATP production has been regarded as the key metabolic mechanism underlying DPN.^[^ ^1^, ^13^, ^25^ ^]^ However, the exact mechanisms leading to ATP reduction have not been well defined, especially in SCs.^[^ ^13a^ ^]^ Herein, we provide new mechanistic insight into how ATP reduction occurs in SCs following DM: rather than a direct ATP reduction, SCs undergo metabolic remodeling to significantly enhance catabolic activity, resulting in a compensatory supply of ATP and enabling the SCs and axons to better withstand adverse external conditions; however, such metabolic remodeling also results in the activation of the CB_1_R‐Hmgcs2 axis, triggering the aberrant ketogenic pathway; consequently, this maladaptive ketogenesis not only diminishes energy metabolism efficiency, but also induces excessive local ketone body accumulation, greatly affecting mitochondrial function and ultimately resulting in ATP reduction, homeostatic decompensation, and myelin disruption. We further showed that the genetic knockout of SC‐CB_1_R can induce metabolic reprogramming of SCs, leading to the blockage of the ketogenic pathway, restoration of metabolic homeostasis, and improvement of neuropathic symptoms. In addition, we explored a way to pharmacologically manipulate SC metabolic remodeling by targeting peripheral CB_1_R, thereby advancing the clinical translation of the above findings.

Previous studies have revealed an extensive involvement of CB_1_R in the regulation of CNS metabolism. Bénard et al. discovered the localization of CB_1_Rs on neuronal mitochondria and showed that such mitochondrial CB_1_Rs regulate neuronal energy metabolism through the modulation of complex I activity, cAMP levels, and PKA activity.^[^ ^26^ ^]^ Further study demonstrated that CB_1_Rs are greatly involved in the regulation of some high brain functions, such as memory and food intake, by modulating mitochondrial activity.^[^ ^5a,d^ ^]^ Other studies found that astrocytic CB_1_R is also responsible for metabolic homeostasis of the CNS and further participates in the regulation of cognitive processes.^[^ ^27^ ^]^ In the present study, we showed that CB_1_R also participates in PNS metabolism and contributes to PNS metabolic disorders by affecting mitochondrial function. Collectively, the aforementioned studies revealed a key role of CB_1_R in modulating nervous system (both central and peripheral) metabolism and its involvement in the physiological functions and pathological alterations of the nervous system, thereby promoting CB_1_R as a critical target to manipulate nervous system activity.

Accumulating studies have reported that ketone bodies, traditionally recognized as energy carriers from the liver to the peripheral tissues, may play beneficial roles in various physiological and pathological conditions. These include, but are not limited to, the suppression of oxidative stress, promotion of myocardial regeneration, renoprotection, alleviation of chemotherapeutic complications, and suppression of tumor growth.^[^ ^28^ ^]^ The role of ketone bodies as signaling metabolites in the regulation of metabolic balance across multiple organs has further been demonstrated.^[^ ^23^, ^29^ ^]^ In the PNS, ketone receptors (like HCAR2) were found in SCs, DRG neurons, and satellite glial cells, and participated in the regulation of neuropathic pain.^[^ ^30^ ^]^ However, the involvement of ketogenesis, particularly peripheral ketogenesis, in various metabolic disorders remains poorly understood. In the present study, we demonstrated that peripheral organs, such as peripheral glial cells, may be endowed with considerable ketogenic capacity under diabetic conditions. Unlike hepatic ketogenesis, peripheral organs cannot accommodate substantial ketogenesis, resulting in the disruption of energy metabolism and the local accumulation of ketone bodies, ultimately leading to homeostatic decompensation. These findings provide new insights into the mechanisms by which maladaptive peripheral ketogenesis underlies metabolic diseases.

In the present study, two key proteins closely associated with CB_1_R were identified in diabetic sciatic nerves by quantitative proteomic sequencing (Figure 3G), including Hmgcs2 and cartilage oligomeric matrix protein (COMP). Hmgcs2, also known as 3‐hydroxy‐3‐ methylglutaryl‐CoA synthase 2, which catalyzes the synthesis of 3‐hydroxy‐3‐methylglutaryl‐CoA (HMG‐CoA) from acetoacetyl‐CoA and acetyl‐CoA, is the first rate‐limiting enzyme in ketogenesis.^[^ ^7^, ^31^ ^]^ Multiple studies further identified Hmgcs2 as a key gene upregulated in the myocardium after DM which is highly associated with diabetic cardiomyopathy.^[^ ^32^ ^]^ It has also been reported that upregulated expression of Hmgcs2 in the kidney and corpus cavernosum plays a role in diabetic kidney disease and DM‐induced erectile dysfunction.^[^ ^33^ ^]^ This evidence indicates that Hmgcs2 plays a key role in the metabolic alterations underlying various diabetic complications. In contrast, COMP, also known as TSP5, is an extracellular matrix protein predominantly expressed within the ligaments and cartilage, which plays an important role in chondrogenesis and tendon/ligament‐related diseases.^[^ ^34^ ^]^ However, the relationship between COMP and metabolic diseases, particularly diabetic complications, has rarely been investigated. Therefore, the primary focus of this study was Hmgcs2. However, the potential involvement of COMP in DPN could not be excluded.

In this study, we primarily focused on maladaptive peripheral ketogenesis and the amelioration of DPN by blocking this process. Nevertheless, there are several issues in other aspects that may need discussion and further investigation. First, PLP‐Cre^ERT2^ mice were employed to achieve targeted manipulation of SCs.^[^ ^35^ ^]^ However, PLP‐Cre^ERT2^ mice also target genes in oligodendrocytes. We provided several lines of evidence that may rule out potential effects from oligodendrocytes: 1) the behavioral baselines of cKO mice were assessed within two weeks before STZ injection (Figure S1F, Supporting Information), thereby excluding potential influence of genetic manipulation on these behavioral tests; 2) JD5037, as a peripherally restricted CB_1_R antagonist with low central occupancy,^[^ ^36^ ^]^ produced behavioral outcomes similar to those of CB_1_R*^fl/fl^*; PLP‐Cre^ERT‐2^ mice; and 3) a large number of histological evaluations of the sciatic nerve were not affected by oligodendrocytes, indicating that the effects of PLP‐CB_1_R manipulation may primarily be confined to the PNS. However, further research may be necessary to completely rule out the influence of oligodendrocyte CB_1_R on behavior under diabetic conditions. Second, the upregulated expression of glycolytic and lipolytic enzymes after DM (Figure 1D,E) and increased acetyl‐CoA level after blocking aberrant ketogenesis (Figure 5E) indicated robust catabolic activity in the diabetic nerves. Given that SCs transport glucose and fatty acids from the extracellular space via insulin‐independent glucose transporters (such as GLUT1) and fatty acid transporters (such as CD36), SCs (both myelinating and non‐myelinating) may obtain acetyl‐CoA through both substantial glycolysis and fatty acid beta‐oxidation.^[^ ^2^, ^37^ ^]^ Especially for myelinating SCs, the degradation of myelin lipids may constitute a significant source of acetyl‐CoA and could be implicated in the etiology of neuropathy.^[^ ^38^ ^]^ However, the exact contribution of glycolysis and lipolysis has not been defined by metabolic flux analysis. Whether glycolysis is actually enhanced or vigorous lipolysis compensates for the lack of glycolysis may require further exploration.

Overall, our study uncovered a new mechanism of maladaptive peripheral ketogenesis in SCs as a contributor to DPN and proposed SC‐CB_1_R as a promising therapeutic target and JD5037 as a pharmacological intervention to reprogram SC metabolism. For future studies, although Tam et al. have characterized the low central occupancy of JD5037 in rodents and its off‐target effects have been rare reported,^[^ ^36^ ^]^ making JD5037 a promising peripherally restricted CB_1_R antagonist, its efficacy and safety profile in human patients with DPN, including potential off‐target effects and limitations for long‐term use, warrant further investigation. On the other hand, it will be interesting to explore the potential roles of maladaptive ketogenesis in more diabetic complications and other metabolic disorders.

## 4 Experimental Section

### Animals

All animal procedures were approved by the Animal Ethics and Welfare Committee of Jilin University. The housing conditions for the mice included a maximum of five individuals per cage, with a 12 h light/dark cycle and unrestricted access to water and rodent feed (Diet 7097, Harlan Teklad). The ambient temperature in the rearing facility was maintained at 23 °C. All genetic mice used for this study were on a C57BL6 background. CB_1_R‐flox mice were donated by Prof. Feng Wang, the University of Chinese Academy of Science; ROSA‐26 td tomato mice were donated by Prof. Weixiang Guo, the Institute of Genetics and Developmental Biology, Chinese Academy of Sciences; and PLP‐CreERT2 mice were obtained from OBiO Technology (C001031). Wildtype male C57BL/6 mice were acquired from Changsheng Biotechnology.

### Key Resources

Key resources including antibodies, sequences, chemicals, peptides, recombinant proteins, and reagents were listed in Tables S1–S5 (Supporting Information).

### Genotyping

Genotyping was performed via PCR amplification of genomic DNA. DNA was isolated from the toes of young mice within 7 days after birth by incubation in 75 ul Lysis Buffer containing 25 mM NaOH and 0.2 mM EDTA for at least 1 h at 98 °C under constant shaking. Then the temperature of mixture was reduced to 15 °C, and the same volume of buffer (40 mM Tris HCl, pH 5.5) was added. The liquid was mixed and used for subsequent PCR assays.

### Animal Model

All experiments were conducted in adult (8 weeks old, 18–22 g weight) male mice. Animals were randomly assigned to each experimental group. Mice were fasted for 24 h before intraperitoneal injection of STZ (180 mg kg^−1^) which was dissolved in 10 mmol L^−1^ sodium citrate buffer (pH = 4.2), with sodium citrate buffer along as control. Blood glucose was measured by blood glucose meter (Roche) one week after STZ injection, and individuals with random blood glucose equal to or greater than 16.7 mmol L^−1^ were included in the subsequent experiment.^[^ ^39^ ^]^

### Tamoxifen Injection

PLP‐Cre^ERT‐2^; rosa‐26 td tomato, CB_1_R*^fl/fl^*; PLP‐Cre^ERT‐2^, and their littermate control (CB_1_R*^fl/fl^*) mice received i.p. injections with tamoxifen (100 mg k^−1^g, dissolved in corn oil and ethanol) every other day for three times.^[^ ^17^ ^]^

### JD5037 Administration

JD5037 was dissolved in a solution containing 10% DMSO, 40% PEG300, 5% Tween80, and 45% saline. Mice in DM + JD and DPN + JD groups were administered with JD5037 (3 mg kg d^−1^) via oral gavage. Mice in MD + Veh group received the same volume of vehicle solution.

### Behavioral Tests

For von Frey test, mice were individually placed in chambers positioned on a wire mesh 55 cm above the table and allowed to acclimate for 45 min. Mouse hind paws were stimulated with a series of von Frey filaments (0.16–1.4 g, Stoelting) to the central plantar surface perpendicularly. The 50% paw withdrawal threshold was determined by the Dixon's up‐down method.^[^ ^40^ ^]^

For hot plate test, the hot plate (Ugo Basile, 35 100) was maintained thermostatically at a temperature of 55 °C. The hind‐paw licking, shaking, or jumping was retained as criteria. A 30s cut‐off was imposed to prevent tissue damage.

For tail‐flick test, the distal end of the mouse's tail was immersed in a water bath maintained at a temperature of 52 °C, and the latency period for tail withdrawal was measured. A 15s cut‐off was imposed as a protection against tissue damage.

### Electrophysiology

The MNCV and SNCV were assessed using the Portable Medical Electromyographic Evoked Potentiometer system (Haishen, NDI‐097). Mice were anesthetized and placed on a 37 °C heating pad to maintain constant body temperature. For MNCV evaluation, a pair of stimulating needle electrodes were placed subcutaneously at the sciatic notch for proximal stimulation and above the ankle for distal stimulation. The recording electrode was placed in the lower foot muscle, between the second and third walking pad. The grounding electrode was placed at the tail. The MNCV was determined by measuring the latency of the proximal and distal recordings, and the distance between the stimulating electrodes, which was measured along the skin surface while maintaining full extension of the lower limbs. For SNCV evaluation, the stimulating electrode was placed in the lower foot muscle, between the second and third walking pad, while the recording electrode was placed subcutaneously at the proximal end of the sciatic notch. The SNCV was determined by measuring the latency and distance from the stimulating to the recording electrode.

### Twitch and Tetanic Force Measurement

Mice were anesthetized and placed on a 37 °C heating pad. The left foot was secured to the foot plate attached to the instrument (Aurora Scientific, 1300A) while the left knee was gently stabilized with the knee clips to ensure its stability. The angle of the foot plate was set at a default value of 17° but could be adjusted for optimal twitch force. Muscle stimulation was achieved through direct stimulation using two needle‐like electrodes with a current intensity of 2 mA. The stimulus pulse width remained constant at 0.2 ms throughout all experiments. Tetanic contractions were induced by delivering stimuli lasting 300 ms at frequencies of 50, 100, and 125 Hz respectively. A resting interval of 2 min separated each tetanic contraction session. Twitch and tetanic contraction forces were normalized based on body weight.^[^ ^41^ ^]^

### βOHB Treatment

βOHB was assisted in dissolving in PBS using ultrasonication, then sterilized by filtering through a 0.22 µm filter membrane, and added to the culture medium in proportion (prepared freshly for immediate use).

### GW6471 Treatment

GW6471 is a potent PPARα antagonist. A stock solution of the compound was prepared at a concentration of 100 mM by dissolving it in DMSO according to the instructions, and was stored in a −80 °C freezer. For use, it was diluted to a working concentration of 10 µM and added to the culture medium for incubation for 24 h.

### Ex Vivo Study on Isolated Sciatic Nerve Explants

The sciatic nerves of adult mice were isolated and the epineurium and perineurium were stripped using micro‐forceps. The explants were incubated in 1 ml ex vivo medium (DMEM/F12 containing 17.5 mM Glucose, 0.5 mM Pyruvate, 1X GlutaMAX, 1X Penicillin/Streptomycin, and 1X N2 supplement) supplemented with βOHB/HG for 6 days in vitro at 37 °C and 5% CO_2_. The medium was changed every 3 days. Samples were then subjected to immunofluorescence staining.

### Immunofluorescence Staining

Mice were deeply anaesthetized with isoflurane and perfused with PBS followed by 4% paraformaldehyde (PFA). For sciatic nerve and DRG staining, the sciatic nerves and DRGs were isolated, fixed in 4% PFA at 4 °C overnight, and dehydrated in 30% sucrose at 4 °C for 24–48 h. Sections of 12 µm were obtained using a cryostat microtome (Leica, CM1950). The slices were washed with PBS, blocked with 5% donkey/goat serum and 0.5% Triton X‐100 for 2 h at room temperature and then primary antibodies in blocking buffer at 4 °C overnight. Subsequently, the slices were washed with PBST (with 0.5% Tween 20), and then incubated with fluorochrome‐conjugated secondary antibodies for 2 h at room temperature. Samples were washed and mounted using coverslips. Images were acquired using a laser‐scanning confocal microscope (Nikon, A1R).

For staining of the intraepidermal nerve fibers, the whole layer of plantar skin was isolated by biopsy punch. After fixation (4% PFA) and sucrose gradient dehydration, sections (50 µm) were obtained by cryostat microtome (Leica, CM1950). After blocking, PGP9.5 was used as the primary antibody for staining, and the remaining procedures were the same as above. The number of nerve fibers crossing the derma‐epidermal junction was calculated as Beiswenger et al. described,^[^ ^42^ ^]^ and the IENFD was expressed as the number of fibers per millimeter.

For neuromuscular junction staining, the extensor digitorum longus was isolated, fixed with 4% PFA at 4 °C overnight, washed with 0.1 M glycine in PBS for 30 min, and incubated with blocking buffer (2% Triton X‐100, 5% BSA, and 5% goat serum) for 2 h at room temperature. After blocking, muscles were incubated with primary antibodies in blocking buffer overnight at 4 °C. The secondary antibodies were incubated overnight at 4 °C after three times of washing. Images were acquired using a laser‐scanning confocal microscope (Nikon, A1R).

### CTB Injection and Staining

Mice received an intramuscular injection of CTB (3 µl, 0.25 mg ml^−1^) into the tibialis anterior and extensor digitorum longus muscle. After a seven‐day period, the animals were subjected to perfusion and fixation. The L3‐5 DRGs were then isolated and prepared for immunofluorescent staining. Confocal microscopy (Nikon A1R) was utilized to capture images of the stained DRG sections. The quantification of CTB‐positive neurons was performed using ImageJ analysis software.

### Transmission Electron Microscopy (TEM)

Mice were euthanized and sciatic nerves were removed and fixed in 2.5% glutaraldehyde at 4 °C overnight, followed by postfixation with 1% osmium tetroxide. The samples were rinsed with phosphate buffer for an additional 10 min, dehydrated through a series of ethanol solutions, and embedded using epoxy resin. Ultrathin sections of the embedded tissues were prepared using an ultramicrotome (LEICA EM UC7). Ultra‐thin sections were placed on a copper grid and stained with 1% uranylacetate and lead citrate. TEM observations and imaging were conducted using a JEOL microscope. For TEM analysis, myelin sheath thickness was determined by g‐ratio analysis by dividing the axonal diameter by the fiber diameter (diameter of axon including the myelin sheath).

### Tissue and Blood Serum Collection for Metabolic Evaluations

Mice were deeply anaesthetized and euthanized by cervical dislocation. The sciatic nerve and its terminals were isolated to the greatest extent possible. The epineurium was immediately trimmed off with micro‐scissors for further investigations. Blood was promptly collected via cardiac puncture using a syringe. After 30 min, serum was obtained through centrifugation at 12 000 rpm for 15 min.

### βOHB Assessment

The relative levels of βOHB in tissue, plasma, and cells were quantified using the Ketone Body detection kits (AAT Bioquest, 138 306). All operating procedures were strictly performed according to the manufacturer's protocol. Briefly, 50 µl of lysates per well were prepared and added to each well of a clear wall 96‐well plate according to the instructions of the kit. This plate was then incubated for 20 min at room temperature. The analysis was performed using a Bio‐Tek Cytation5 instrument.

### ATP Assessment

The relative levels of ATP in tissues and cells were quantified using the ATP assay kit (Beyotime, S0026), which facilitates cell lysis and generates a luminescent signal that was proportional to the ATP concentration. In brief, 20 µl lysates were prepared and added to individual opaque wells of 96‐well plate in accordance with the kit instructions. The plate was then incubated at room temperature for 20 min. The analysis was conducted using a Bio‐Tek Cytation5 instrument.

### siRNA Interference

For RNA interference, CB_1_R siRNA, Hmgcs2 siRNA, and negative control siRNA (Integrated Biotech Solutions) were transfected using Lipofectamine 3000 reagent (Invitrogen, L3000150). After 6 h of transfection, the medium was replaced with fresh DMEM/F12 medium containing 10% FBS for additional culture of 72 h. The negative control was designed as a nonspecific sequence.

### Cell Cultures

For primary Schwann cells culture, sciatic and brachial plexus nerves were isolated from 2‐day‐old rats, minced, and incubated with 3 mg mL^−1^ collagenase (Sigma, C0130) for 30 min at 37 °C, followed by incubation with trypsin (Sigma, T4049) for 8 min at 37 °C. Cells were maintained in DMEM/F‐12 medium supplemented with 10% FBS, 100 IU mL^−1^ penicillin, and 100 g mL^−1^ streptomycin at 37 °C in a 5% CO_2_ humidified atmosphere. Cells were then treated with 10 µM cytosine β‐D‐ara‐furan (Sigma–Aldrich, C1768), polyclonal anti‐Thy1.1 antiserum (1:1000, Sigma–Aldrich, M7898), and rabbit complement (Millipore, 234400) to remove fibroblasts. Primary neurons were obtained from the DRGs of 6‐8‐week‐old rats. In brief, the DRGs were isolated and digested using collagenase/trypsin (Sigma, C0130, T4049). Neurons were then seeded in poly‐L‐lysine (Sigma, P8954) coated 24‐well plates. 24 h later, the medium was replaced with DMEM/F12 or HG (75 mM) medium supplemented with B‐27.

Rat brain endothelial (RBE4) cells (Shanghai boke Biotechnology, E0423) were cultured in 6‐cm dishes or in 6‐well plates containing DMEM/F12 medium supplemented with 10% FBS, 100 IU mL^−1^ penicillin, and 100 g mL^−1^ streptomycin and grown in a 5% CO_2_ atmosphere at 37 °C.

### Mitochondrial Morphology Evaluation

Cells were seeded in confocal dishes (NEST, 801 001) and loaded with 100 nM Mito‐Tracker Green Probes (Beytime, C1048) at 37 °C for 30min. The cells were photographed using a confocal microscope with oil lens objective. The images were analyzed using ImageJ software.^[^ ^43^ ^]^

### Mitochondria Membrane Potential Assessment

To monitor mitochondrial health, JC‐1 dye (Beyotime, C2006) was used to assess the mitochondrial membrane potential. Cells were seeded in confocal dishes and subjected to different treatment protocols. JC‐1 probes at a concentration of 1 uM were loaded and incubated at 37 °C in the absence of light for 30 min. Images were captured using a confocal microscope immediately after 3 rounds of washing.

The mitochondrial membrane potential was also evaluated through flow cytometry. The cells were seeded in 6‐well plates and then collected after different treatments. Subsequently, 1 uM JC‐1 probes were added and incubated at 37 °C in the absence of light for 30 min. After washing, the mitochondrial membrane potential was analyzed through flow cytometry (BD LSR Fortessa) and FlowJo software.

### Intracellular ROS Evaluation

Cells seeded in confocal dishes or 6‐well plates were subjected to different treatment protocols. 3 uM DCFH‐DA probes (Beyotime, S0033s) were added and incubated at 37 °C for 30 min. The images were acquired using a confocal microscope or flow cytometry. The average fluorescence intensity was quantified using ImageJ software, and the intracellular ROS levels were analyzed by FlowJo software.

### Mitochondrial ROS Production

The mitochondrial ROS indicator, MitoSOX Red (Invitrogen, M36005), was used to evaluate mitochondrial ROS production. Cells were incubated with MitoSOX (5 µM) at 37 °C for 20 min following the manufacturer's protocol. The images were acquired using a confocal microscope and the average fluorescence intensity was quantified using ImageJ software.

### Cell Viability Assay

The cell viability was assessed using the Cell Counting Kit‐8 (CCK‐8) assay. SCs were seeded in 96‐well plates and treated with different concentrations of βOHB as described above for 24 h. Subsequently, 10 µl of CCK‐8 reagent (DoJinDo) was added to each well and incubated in the dark at 37 °C for 2 h. Optical density values (OD450 nm) were measured using a microplate reader.

### Real‐Time qPCR Analysis

Total RNA was extracted using the Eastep Super Total RNA Extraction Kit (Promega, LS1040). cDNA synthesis was performed using the transcript One‐Step cDNA Synthesis SuperMix (TransGen Biotech, AT311). Primers were synthesized by Genewiz Biotech as listed above. The cDNA template and primers were combined with TB Green Premix Ex Taq (TaKaRa, RR420A) for quantitative real‐time PCR analysis using a Real‐time PCR system (Bio‐Rad, CFX96). Relative expression levels were calculated using the 2^−ΔΔCt^ method. β‐actin served as loading control.

### Western Blot Analysis

Primary SCs or sciatic nerves were lysed in RIPA buffer supplemented with 1 × protease inhibitor mixture (Sangon Biotech, C50008). The protein concentrations were quantified utilizing the BCA assay (Beyotime Biotechnology, P0010). Protein samples were then separated by 10% SDS‐PAGE and transferred onto PVDF membranes. Subsequently, the membranes were blocked for 1.5 h at room temperature and incubated overnight at 4 °C with primary antibodies. Membranes were incubated with secondary antibodies (1:1000, Beyotime) for 2 h at room temperature. After completing the membrane development exposure, we wash off the excess developing solution with TBST, and then immerse the membrane in the Western Blot Antibody Stripping Solution (Epizyme) at room temperature for 20 min to elute the antibodies. Subsequently, it was added TBST to remove the stripping solution, and then re‐block and incubate with antibodies as described in the aforementioned steps. Images were scanned using a GS800 densitometer and analyzed using PD Quest 7.2.0 software. Band density was normalized to loading control. The experiment was repeated three times and representative images were presented.

### ELISA

ELISA acetyl‐CoA Assay Kit was used to quantify A‐CoA levels in mouse tissues and cells according to the manufacturer's protocols.

### Mitochondrial Citrate Acid

Cells were treated with acidic lysate, thoroughly homogenized, and centrifuged at 600 g for 5 min. The resulting precipitate was mixed with acidic extract, followed by ultrasonic disruption. Subsequently, an equal volume of alkaline extract was added and mixed. Finally, the mixture was transferred into a UV plate (96‐well) for detection using a microplate reader.

### Protein Digestion and TMT Labeling

Samples were washed with PBS in an Eppendorf (EP) tube, followed by the addition of protease and trypsin for digestion. The resulting tryptic peptides were then transferred to a 1.5 mL tube to terminate the digestion process. After confirming the pH of the samples, desalting was performed using the SOLAμ system (Thermo Fisher Scientific). Subsequently, the peptides were labeled with the TMTpro 16 plex Isobaric Label Reagent Set. The peptides were separated using high‐performance liquid chromatography (HPLC) and combined into 30 fractions. After that samples were sent for LC‐MS analysis.

### LC‐MS Analysis

The liquid chromatography‐tandem mass spectrometry (LC‐MS/MS) analysis was conducted using a nanoflow DIONEX UltiMate 3000 RSLCnano System, which was interfaced with an Orbitrap Exploris 480 mass spectrometer (Thermo Scientific), and this setup included a FAIMS Pro device (Thermo Scientific). The system operated in data‐dependent acquisition (DDA) mode.

### Statistical Analysis

All data are shown as mean ± s.e.m. Sample size was determined based on the basis of previous experiments using similar methodologies. Data were analyzed by GraphPad Prism 10.0. Statistical comparisons were made using two‐tailed *t* test, one‐way ANOVA, or two‐way ANOVA as indicated in the figure legends. Differences were considered significant when *p* < 0.05. Asterisks correspond to the following significance levels: ns, not significant, *p < 0.05, **P < 0.01, ***P < 0.001, and ****P < 0.0001.

## Conflict of interest

The authors declare no conflict of interest.

## Author Contributions

W.L. and T.Y. contributed equally to this work. W.L., T.Y., R.C., and C.S. performed conceptualization; W.L., T.Y., N.W., C.M., K.Y., and X.Z. performed investigation; W.L. and T.Y. performed formal analysis; W.L. and T.Y. wrote the original draft; R.C. and C.S. performed supervision; W.L., T.Y., and B.L. performed methodology; R.C. and C.S. performed project administration; R.C. and C.S. performed funding acquisition.

## Supporting information



Supporting Information

## Data Availability

The data that support the findings of this study are available from the corresponding author upon reasonable request.
