# Supplementary material for: Maladaptive Peripheral Ketogenesis in Schwann Cells Mediated by CB1R Contributes to Diabetic Neuropathy
Source: Adv Sci (Weinh). 2025 Jan 30;12(13):2414547. doi: 10.1002/advs.202414547 (PMC11967812; doi:10.1002/advs.202414547)
Supplement: Supplementary file 1 — Supporting Information [file ADVS-12-2414547-s001.docx]

Supporting Information

**Maladaptive Peripheral Ketogenesis in Schwann Cells Mediated by CB_1_R Contributes to Diabetic Neuropathy**

*Weizhen Li, Tuo Yang, Ningning Wang, Baolong Li, Chuikai Meng, Kaiming Yu, Xiongyao Zhou, Rangjuan Cao*, and Shusen Cui**

This Supporting Information file includes:

Figure S1 to Figure S6

Table S1 to Table S5


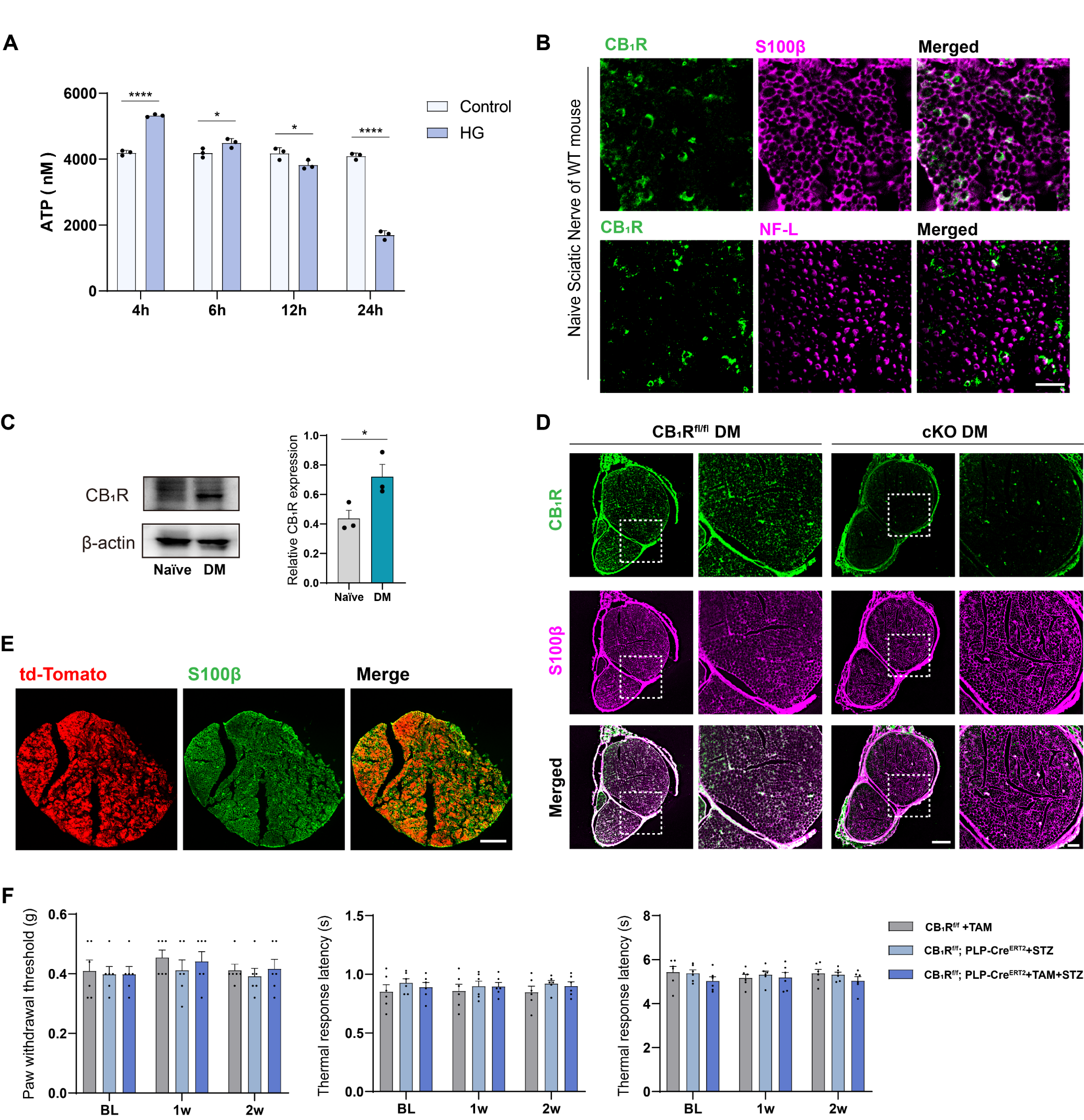


**Figure S1.** Profile of ATP alterations in SCs and validation of the SC-CB_1_R knockout mice. A) ATP levels of primary cultured SCs at 4, 6, 12, and 24 hours after HG treatment. n = 3 per group. B) Representative images of ssCB_1_R, S100β labeled SCs and NF-L labeled axons in the sciatic nerves of WT mice. Scale bar, 20 μm. C) Western blot analyses and quantifications of CB_1_R expression in the sciatic nerve of WT mice. D) The localization of PLP in SCs was determined by immunostaining of S100β in the sciatic nerve of PLP-CreERT2; Rosa26-tdTomato mice. Scale bar, 100 μm. E) The knockout efficiency was determined by immunostaining of CB_1_R and S100β in the sciatic nerve of diabetic cKO mice and diabetic littermate control mice. Representative images and magnification of the sciatic nerve. Scale bar, 100 μm (left), 30 μm (right). F) Behavioral tests within 2 weeks after TAM injection, including Von Frey test (left), hot plate test (middle), and tail flick test (right). n = 6 per group. All data are expressed as mean ± SEM. Statistical comparisons were conducted with two-way ANOVA followed by Bonferroni’s post hoc test (A, F) and two-tailed t-test (C). **P* < 0.05, and *****P* < 0.0001.

**
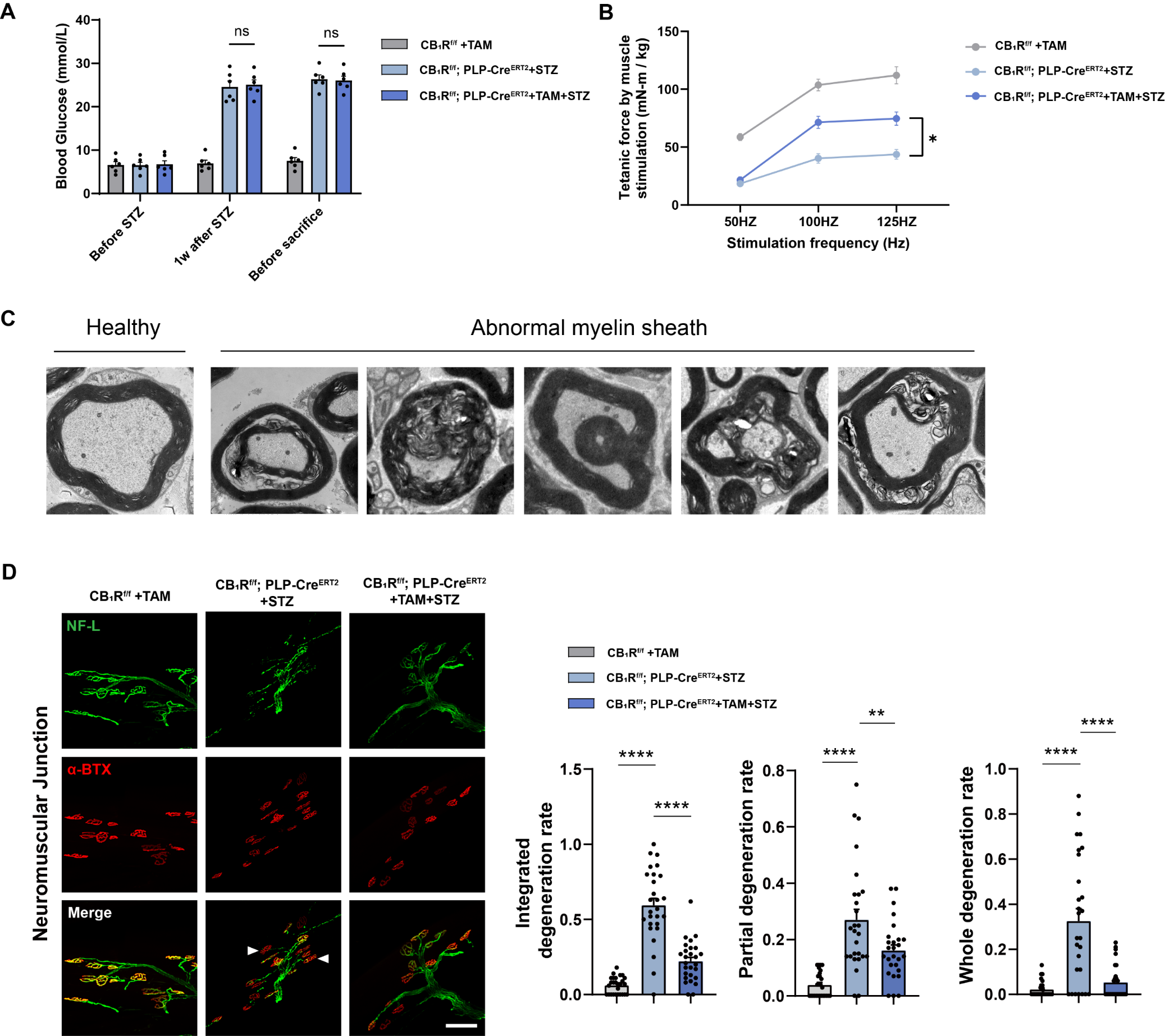
Figure S2.** SC-CB_1_R knockout attenuates DPN. A) Blood glucose levels were assessed prior to STZ injection, 1 week following STZ injection, and before sacrifice. n = 6 per group. B) Tetanic force in reaction to stimulations of 50, 100 and 125 Hz. n = 6 per group. C) Sample graphs of healthy and abnormal myelin sheath. D) representative images and quantifications of NMJ degeneration. Arrows indicate representative degenerated NMJs. Scale bar, 100 μm. n = 6 mice per group. Each data point represents an individual slice. All data are expressed as mean ± SEM. Statistical comparisons were conducted with two-way ANOVA followed by Bonferroni’s post hoc test (A, B); and one-way ANOVA followed by Tukey’s post hoc test (D). **P* < 0.05, ***P* < 0.01, and *****P* < 0.0001.


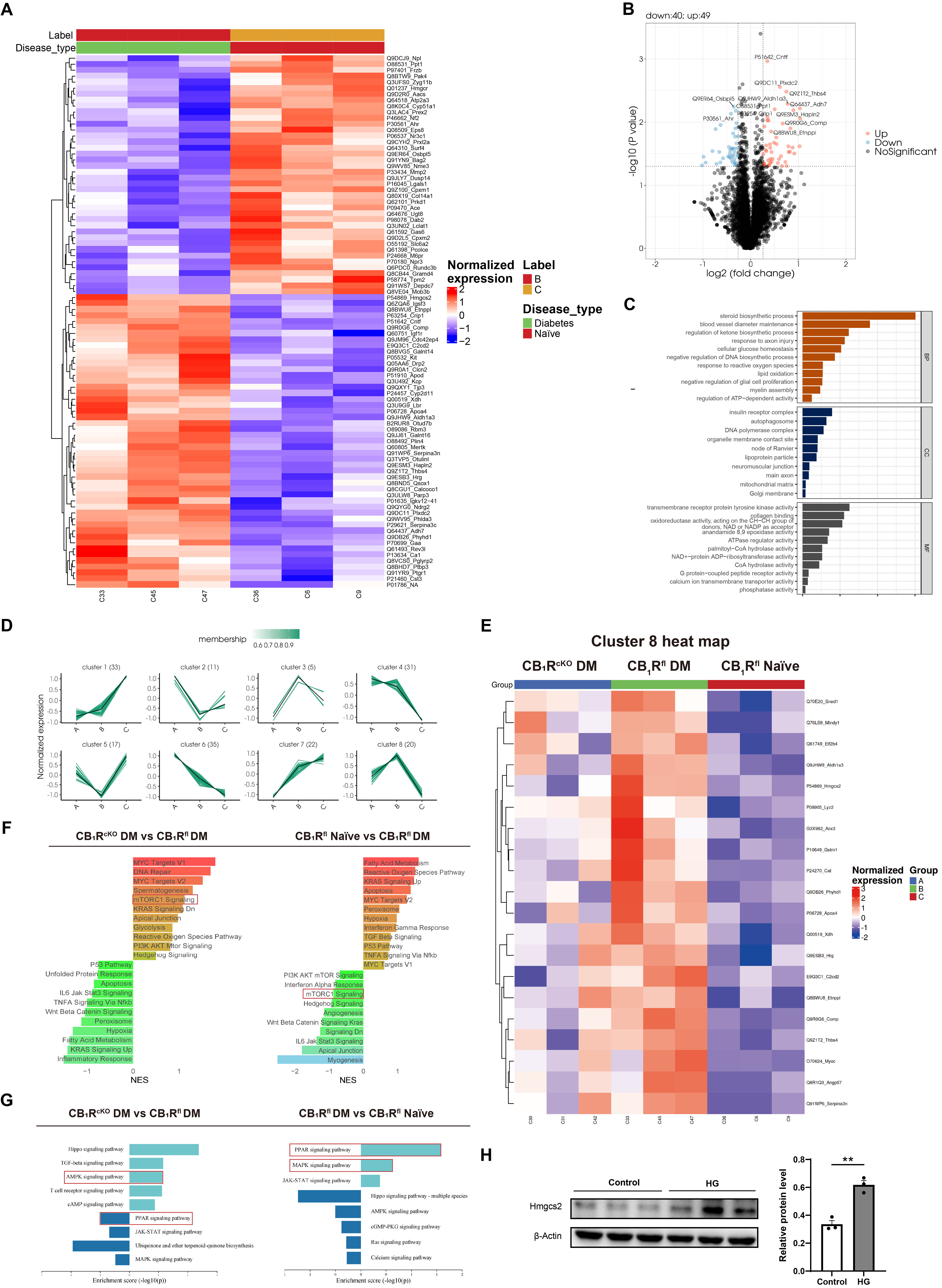


**Figure S3.** Proteomic analysis of the SC-CB_1_R cKO mice. A, B) Heatmap (A) and volcano map (B) of differential proteins between CB_1_R^fl^ Naïve mice and CB_1_R^fl^ DM mice. C) GO analysis of the differential proteins between CB_1_R^fl^ Naïve mice and CB_1_R^fl^ DM mice. D) All quantified proteins were subjected to Mfuzz cluster analysis and divided into 8 clusters based on their expression alterations among the three groups of experimental mice. E) Heatmap illustrating the protein expression profiles within Cluster 8 across all three groups. F) Gene set enrichment analysis indicating statistically significant enrichment of CB_1_R^cKO^ DM vs CB_1_R^fl^ DM as well as CB_1_R^fl^ Naïve vs CB_1_R^fl^ DM. NES, normalized enrichment score. G) KEGG pathway analysis of CB_1_R^cKO^ DM vs CB_1_R^fl^ DM, and CB_1_R^fl^ DM vs CB_1_R^fl^ Naïve. H) Western blot and quantification of Hmgcs2 expression in primary SCs with or without HG, with β-actin indicating loading control. n = 3 per group. All data are expressed as mean ± SEM. Statistical comparisons were conducted with two-tailed t-test (H). ***P* < 0.01.

**
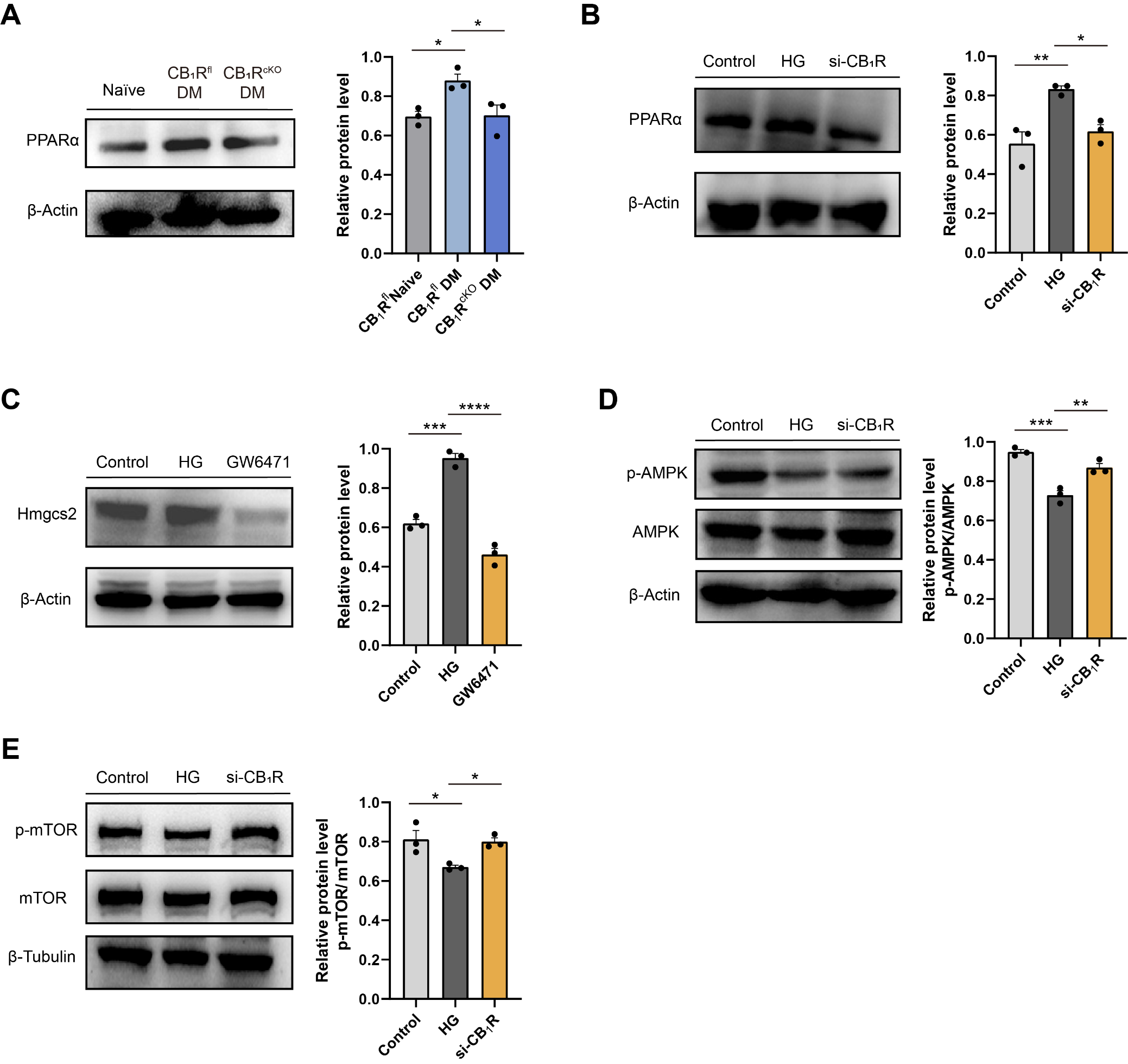
Figure S4.** SC-CB_1_R regulates Hmgcs2 through AMPK-mTOR-PPARα pathway. A) Western blot and quantification of PPARα in the sciatic nerves with β-actin used as a loading control. n = 3 per group. B-E) Western blot and quantification of PPARα (B), Hmgcs2 (C), p-AMPK/AMPK (D) and p-mTOR/mTOR (E) in primary SCs with β-actin or β-tubulin used as a loading control. n = 3 per group. All data are expressed as mean ± SEM. Statistical comparisons were conducted with one-way ANOVA followed by Tukey’s post hoc test. **P* < 0.05, ***P* < 0.01, ****P* < 0.001, and *****P* < 0.0001.


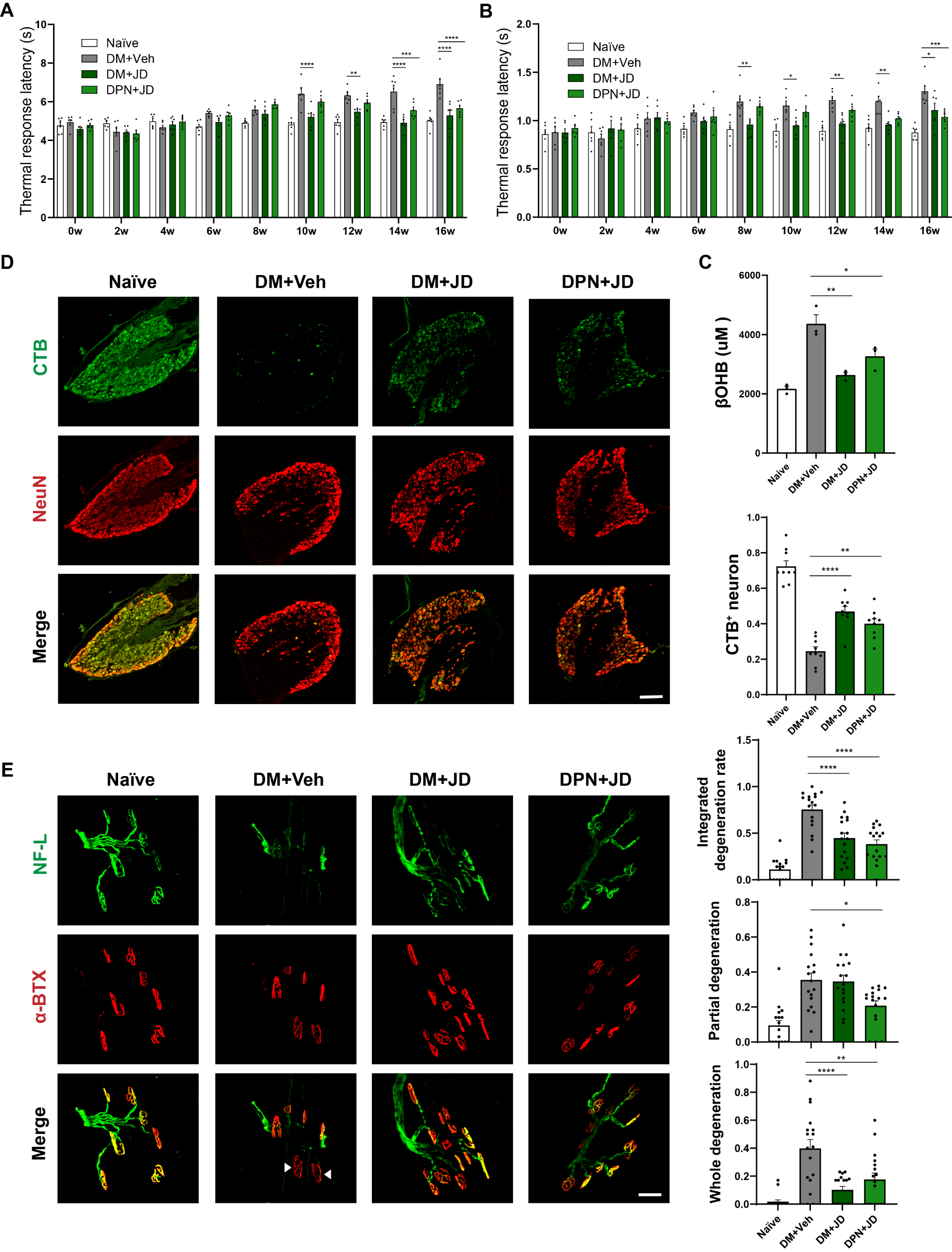


**Figure S5.** JD5037 attenuates DPN. A, B) Behavioral tests including hot plate (A) and tail-flick (B) tests. n = 6 per group. C) βOHB levels in the sciatic nerves 4 months post-DM. n = 3 per group. D) CTB traced neurons were evaluated by immunostaining of Neun in the affected DRGs. Scale bar, 200 μm. n = 9 slices from 3 mice. E) NMJ degeneration was evaluated by immunostaining of NF-L and α-BTX. Scale bar, 50 μm. Arrows indicate representative degenerated NMJs. n = 6 mice per group. Each data point represents an individual slice. All data are expressed as mean ± SEM. Statistical comparisons were conducted with two-way ANOVA followed by Bonferroni’s post hoc test (A, B); and one-way ANOVA followed by Tukey’s post hoc test (C-E). **P* < 0.05, ***P* < 0.01, ****P* < 0.001 and *****P* < 0.0001.


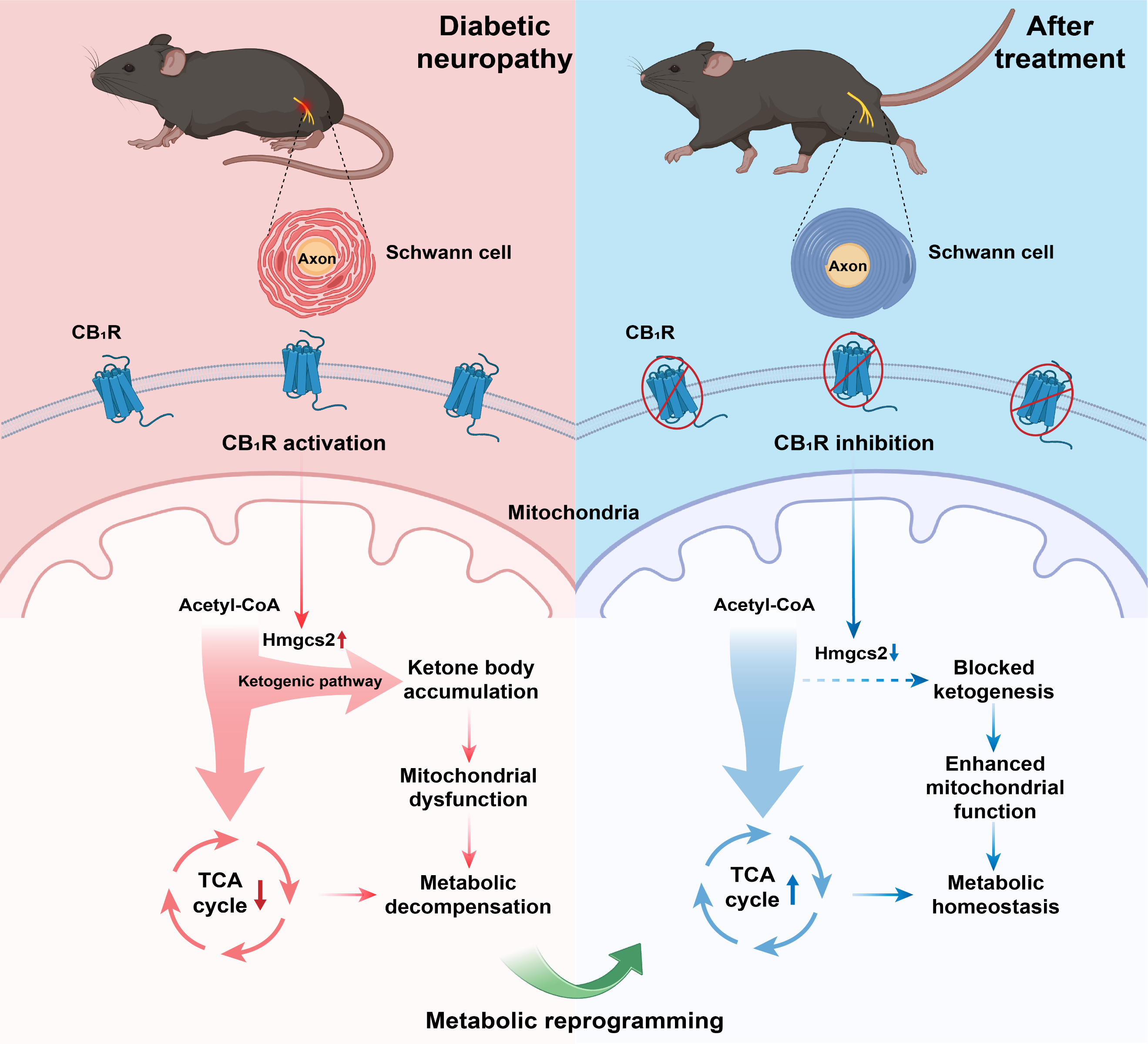


**Figure S6.** CB_1_R inhibition induces SC metabolic reprogramming by blocking maladaptive ketogenesis. The CB1R-Hmgcs2 axis is activated following the onset of diabetes, leading to a metabolic remodeling of SCs involving aberrant ketogenesis. The PNS can hardly adapt to such localized ketogenesis, resulting in disrupted energy metabolism, mitochondrial damage, and homeostatic decompensation, ultimately contributing to DPN. Silencing CB_1_R can induce metabolic reprogramming of SCs by blocking maladaptive ketogenesis, resulting in rebalanced energy metabolism, reduced histopathological changes, and improved neuropathic symptoms.

**Table S1. Antibodies**

| **Antibodies** | **Source** | **Identifier** |
| --- | --- | --- |
| Rabbit Anti-S100β | Abcam | Cat#ab52642 |
| Mouse Anti-S100β | Sigma | Cat#S2532 |
| Rabbit Anti-NFL | Cell Signaling Technology | Cat#2837 |
| Mouse Anti-NF-200 | Sigma | Cat#N5389 |
| Mouse Anti-PGP9.5 | Sigma | Cat#ab1761-I |
| Mouse Anti-Tuj1 | Abcam | Cat#ab78078 |
| Rabbit Anti-ATF3 | Abcam | Cat#ab207437 |
| Rabbit Anti-Synapsin-1 | Cell Signaling Technology | Cat#5297 |
| Mouse Anti-β-Actin | Transgen | Cat#HC201 |
| Mouse Anti-β-Tubulin | Transgen | Cat#HC101 |
| Rabbit Anti-Cannabinoid receptor 1 | Abcam | Cat#ab3558 |
| Rabbit Anti-NeuN | Cell Signaling Technology | Cat#24307 |
| Rabbit Anti-Hmgcs2 | Cell Signaling Technology | Cat#40364 |
| Monoclonal Anti-Thy1.1 | Sigma | Cat#M7898 |
| Rabbit Anti-PPARα | Abcam | Cat#ab126285 |
| Rabbit Anti- Phospho-mTOR (S2448) | ABMART | Cat#T56571 |
| Rabbit Anti- mTOR | ABMART | Cat#T55306 |
| Rabbit Anti- Phospho-AMPKα(Thr-172) | Affinit | Cat#AF3423 |
| Rabbit Anti-AMPKα | Affinit | Cat#AF6423 |
| Alexa Fluor 488 Donkey Anti-Mouse | Invitrogen | Cat#A32766 |
| Alexa Fluor 488 Donkey Anti-Rabbit | Invitrogen | Cat#A11008 |
| Alexa Fluor 546 Donkey Anti-Mouse | Invitrogen | Cat#A10040 |
| Alexa Fluor 546 Donkey Anti-Rabbit | Invitrogen | Cat#A11035 |
| Alexa Fluor 647 Goat Anti-Mouse | Invitrogen | Cat#A31571 |
| HRP-Donkey-anti-rabbit | Beyotime | Cat#A0208 |
| HRP-Donkey-anti-mouse | Beyotime | Cat#A0216 |

**Table S2. qPCR primers**

| **qPCR primer** | **Sequence** |
| --- | --- |
| β-Actin-F | CATTGCTGACAGGATGCAGAAGG |
| β-Actin-R | TGCTGGAAGGTGGACAGTGAGG |
| HK-F | GAAAGGAGACCAACAGCAGAGC |
| HK-R | TTCGTTCCTCCGAGATCCAAGG |
| GPD1-F | CGCATCACTGTGGTACAAGAGG |
| GPD1-R | CTTGGTGTTGTCACCGAAGCCA |
| PFK1-F | CTGTTCGCTCTACCGTGAGGAT |
| PFK1-R | TTGGAACCACCTTGACCAGTCC |
| CPT1-F | GGCATAAACGCAGAGCATTCCTG |
| CPT1-R | CAGTGTCCATCCTCTGAGTAGC |
| CPT2-F | GATGGCTGAGTGCTCCAAATACC |
| CPT2-R | GCTGCCAGATACCGTAGAGCAA |
| ACACA-F | GTTCTGTTGGACAACGCCTTCAC |
| ACACA-R | GGAGTCACAGAAGCAGCCCATT |
| FASN-F | CACAGTGCTCAAAGGACATGCC |
| FASN-R | CACCAGGTGTAGTGCCTTCCTC |
| BDH1-F | AGGCTGTGACTCTGGATTTGGG |
| BDH1-R | CTGGATGGTTCTCAGTCGGTCA |
| Hmgcl-F | GCACTTTGCCAAAGCAGGTGAAG |
| Hmgcl-R | CGGAAAGCATGTCGATCAGCCT |
| Oxct1-F | GAGCGACAGTTCCTTTCTGGTG |
| Oxct1-R | TCCCATACCCTGTGCTGGTGTA |
| Hmgcs2-F | TGCTATGCAGCCTACCGCAAGA |
| Hmgcs2-R | GCCAGGGATTTCTGGACCATCT |
| CS-F | ATGCAGAGGGAATGAACCGAGC |
| CS-R | GAGTCAATGGCTCCGATACTGC |

**Table S3. si-RNA Sequences**

| CB1R | Sense | 5’-GGGAAGAUGAACAAGCUUAUC-3 |
| --- | --- | --- |
|  | Anti-sense | 5’-UAAGCUUGUUCAUCUUCCCGA-3’ |
| Hmgcs2 | Sense | 5’-GCUGGUGUCUAGUGUGUCAGA-3 |
|  | Anti-sense | 5’-UGACACACUAGACACCAGCUU-3’ |
| Negative | Sense | 5’-UUCUCCGAACGUGUCACGUTT-3 |
|  | Anti-sense | 5’-ACGUGACACGUUCGGAGAATT-3’ |

**Table S4. Chemicals, peptides, recombinant proteins, and Reagent Resource**

| JD5037 | MedChemExpress | Cat#HY-18697 |
| --- | --- | --- |
| GW6471 | MedChemExpress | Cat# HY-15372 |
| DMED/F12 | Bioscience | Cat#3130-0500 |
| Corn Oil | MedChemExpres | Cat#HY-Y1888 |
| Opti-MEM | Gibco | Cat#31985-070 |
| Streptozotocin | Sigma | Cat#S0130 |
| βOHB | MedChemExpress | Cat#HY-113378 |
| Tamoxifen | MedChemExpress | Cat#HY-13757A |
| α-Bungarotoxin | BTPROBES | Cat#90-1022 |
| Cholera toxin subunit B | Absin | Cat#abs80003 |
| DAPI | Genview | Cat#GD3410 |
| Hochest 3342 | Solarbio | Cat#23491 |
| Cytosine β-D-arabinofuranoside-crystalline | Sigma | Cat#C1768 |
| Poly-L-lysine Hydrobromide | Sigma | Cat#P8954 |
| Forskolin | Sigma | Cat#F6886 |
| Recombinant Human NRG1-β1 | R&D | Cat#377-HB-050 |
| Collagenase from Clostridium histolyticm | Sigma | Cat#C0130 |
| Complement, Rabbit Serum | Calbiochem | Cat#3386909 |
| Fetal Bovine Serum | Sigma | Cat#F8318 |
| 1XPBS | Bioss | Cat#C01-01001 |
| 1XTBST | Servicebio | Cat#G2150 |
| NGF | Sino Biological | Cat#11050-HNAC |
| Protease inhibitor mixture | Sangon Biotech | Cat# C50008 |
| B27 | Gibco | Cat#17504044 |
| N2-supplyment | Gibco | Cat#17502-048 |
| GlutMAX | Gibco | Cat#35050061 |
| Pyruvate | Sigma | Cat#P2256 |
| MitoSOX Red | Invitrogen | Cat#M36005 |
| Poly-L-ornithine hydrobromide | Sigma | Cat#P3655 |
| Lipofectamine 3000 | Invitrogen | Cat#L3000150 |
| Bovine serum Albumin | Genview | Cat#FA016 |
| Donkey serum | Servicebio | Cat#G1217 |
| Goat serum | Servicebio | Cat#G1208 |
| DMSO | Sigma | Cat#D2650 |
| Penicillin-Streptomycin Solution | Beyotime | Cat#C0222 |
| Mitotracker-Green | Beyotime | Cat#C1048 |
| Tween-20 | Ding Guo | Cat#DH358-3 |
| Tween-80 | Ding Guo | Cat#DH360-3 |
| PEG-300 | Shyuanye | Cat#25322-68-3 |
| TritonX-100 | Ding Guo | Cat#DH351-4 |
| Western Blot Antibody Stripping Solution | Epizyme | Cat#PS107 |

**Table S5. Critical commercial assays**

| Ketone Body Assay Kit | AAT Bioquest | Cat#13830 |
| --- | --- | --- |
| ATP Assay Kit | Beyotime | Cat#S0026 |
| ROS Assay Kit | Beyotime | Cat#S0033s |
| TB Green™ Premix Ex Taq™ | TaKaRa | Cat#RR420A |
| CCK-8 Assay Kit | DoJinDo | Cat#CK04 |
| TaKaRa MiniBEST Universal RNA Extraction kit | TaKaRa | Cat#9767 |
| JC-1 | Beyotime | Cat#C2006 |
| ELISA acetyl-CoA Assay Kit | Elabscience | Cat#E-EL-0125 |
| Mitochondrial Citrate Acid | LMAI Bio | Cat#LM-A457 |
